# Pyrazolo[1,5-*a*]pyrimidines-based fluorophores: a comprehensive theoretical-experimental study†

**DOI:** 10.1039/d0ra07716j

**Published:** 2020-10-29

**Authors:** Alexis Tigreros, Sandra-L. Aranzazu, Nestor-F. Bravo, Jhon Zapata-Rivera, Jaime Portilla

**Affiliations:** Bioorganic Compounds Research Group, Department of Chemistry, Universidad de los Andes Carrera 1 No. 18A-10 Bogotá 111711 Colombia jportill@uniandes.edu.co; Molecular Electronic Structure Group, Department of Chemistry, Universidad de los Andes Carrera 1 No. 18A-10 Bogotá 111711 Colombia

## Abstract

Fluorescent molecules are crucial tools for studying the dynamics of intracellular processes, chemosensors, and the progress of organic materials. In this study, a family of pyrazolo[1,5-*a*]pyrimidines (PPs) 4a–g has been identified as strategic compounds for optical applications due to several key characteristics such as their simpler and greener synthetic methodology (RME: 40–53%) as compared to those of BODIPYS (RME: 1.31–17.9%), and their tunable photophysical properties (going from *ε* = 3320 M^−1^ cm^−1^ and *ϕ*_F_ = 0.01 to *ε* = 20 593 M^−1^ cm^−1^ and *ϕ*_F_ = 0.97), in which electron-donating groups (EDGs) at position 7 on the fused ring improve both the absorption and emission behaviors. The PPs bearing simple aryl groups such as 4a (4-Py), 4b (2,4-Cl_2_Ph), 4d (Ph) and 4e (4-MeOPh), allow good solid-state emission intensities (QY_SS_ = 0.18 to 0.63) in these compounds and thus, solid-state emitters can be designed by proper structural selection. The properties and stability found in 4a–g are comparable to commercial probes such as coumarin-153, prodan and rhodamine 6G. Ultimately, the electronic structure analysis based on DFT and TD-DFT calculations revealed that EDGs at position 7 on the fused ring favor large absorption/emission intensities as a result of the ICT to/from this ring; however, these intensities remain low with electron-withdrawing groups (EWGs), which is in line with the experimental data and allows us to understand the optical properties of this fluorophore family.

## Abstract 2

A family of 7-aryl-3-methylpyrazolo[1,5-*a*]pyrimidines have been identified as strategic compounds for optical applications by a comprehensive theoretical-experimental study.

## Graphical abstract

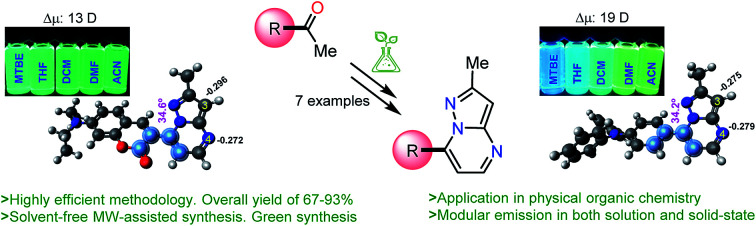

## Introduction

Fluorescent organic compounds have been a major focus of research related to materials science and biological interactions over the past decades.^1^ Ranging from ionic or molecular sensing^2^ to bioimaging applications^3^ and, from the organic light-emitting devices,^4^ bio-macromolecular interactions.^5^ A plethora of applications has been proposed by exploiting the beneficial properties of such materials that can raise our standard of living. In this field, the fluorogenic heterocyclic compounds display advantages over hydrocarbon-based fluorophores such as (i) synthetic access methodologies that allow structural diversity, (ii) heteroatoms (B, N, O or S) that make them potential chelating agents for ions, and (iii) better solubility in green solvents. Consequently, various fluorescent derivatives have been applied in diverse fields, and include coumarins,^6^ pyrazoles,^7^ perylene bisimides,^8^ boron dipyrromethene difluoride (BODIPY),^9^ cyanines,^10^ and rhodamines.^11^

Importantly, for biological and/or optoelectronics applications, ideal fluorescent probes must meet several requirements: the synthetic pathway should be straight (few steps), useful (high yields and low waste generation), and carried out by efficient heating technologies; *i.e.*, microwave (MW) or ultrasound (US). Because a probe for bioimaging is inherently involved in complex biological interactions within the cell, the probe must be as small as possible to reduce its impacts on the biological dynamics and facilitate its diffusion inside the cell organelles.^3^ Likewise, for most of the photophysical applications, the fluorophore must be able to change its absorption and emission properties with small and simple chemical modifications. Fulfilling the above-mentioned conditions in a single fluorophore is a challenging task and many efforts need to be made in this direction. Recently, pyrazolo[1,5-*a*]pyrimidines (PPs) have emerged as an attractive alternative due to their small size based on [5,6]-fused N-heterocyclic systems, their efficient synthetic approaches^12^ and easy functionalization methodologies,^13,14^ together with their fluorescence properties (high quantum yields in different solvents, and excellent photostability).^15^ Usually, the PPs receive major attention in biological applications, with the cancer therapeutics field being the most attractive area.^16^ Very recently, Professor Jian-Feng Ge's group found that the combination of photophysical properties with biological activities allows the use of these compounds as lipid droplet biomarkers for HeLa cells (cancer cells) and L929 cells (normal cells),^15^ demonstrating the interesting versatility of this core.

Among the synthetic procedures available for the preparation of diverse pyrazolo[1,5-*a*]pyrimidine (PP) derivatives,^7,12–21^ the strategy involving the cyclocondensation of NH-3-aminopyrazoles with β-dicarbonyl compounds or other 1,3-bis-electrophiles (*e.g.*, alkoxymethylene-β-dicarbonyl compounds, α,β-unsaturated systems, β-enaminones, β-ketonitriles, β-enaminonitriles, among others) has been the most frequently studied due to its excellent performance. This synthetic approach allows key structural modifications at all the peripheral positions during ring-construction and through subsequent functionalization steps.^7,12–21^

**Scheme 1:**
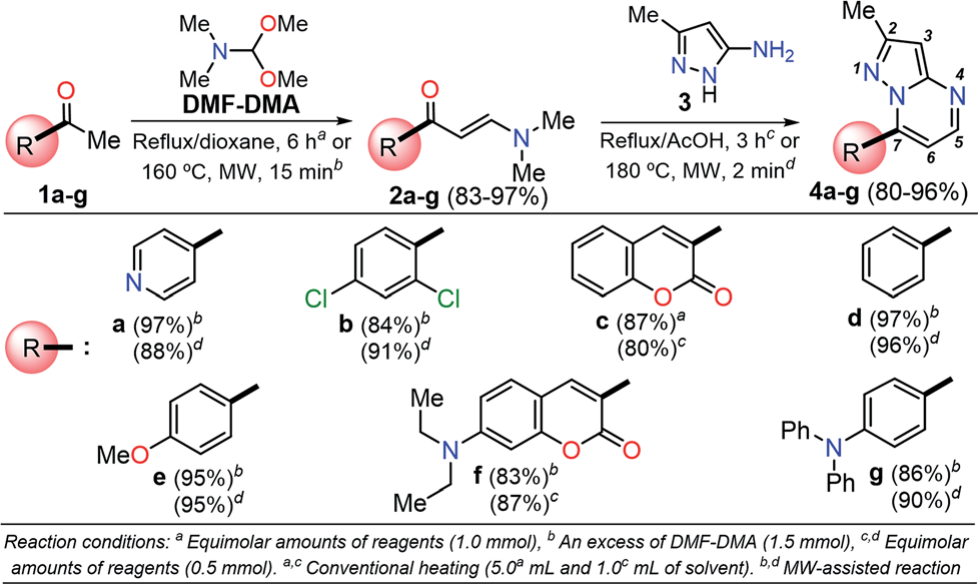
Synthesis of pyrazolo[1,5-*a*]pyrimidine compounds 4a–g.

It is important to note that the theoretical calculations are an important tool for examining the electronic and reactivity properties of some interesting fluorophores.^22–25^ For example, the excited-state intramolecular proton transfer (ESIPT) process,^22^ absorption and emission transitions,^23^ aggregation-caused quenching mechanism,^24^ and other crucial chemical properties of the fluorescent molecules^25^ have been investigated. From this perspective and in line with the aforementioned properties of the pyrazolo[1,5-*a*]pyrimidines, we proposed the synthesis of a family of these fused N-heterocycles (compounds 4a–g) substituted at position 7 with different electron-withdrawing (EWGs) and electron-donating groups (EDGs). The fluorophores 4a–g were obtained by the interaction of the appropriate β-enaminone 2a–g with 3-methyl-1*H*-pyrazol-5-amine (3); their green chemistry efficiency and the cost per gram of raw materials in each case were evaluated (Scheme 1). Likewise, the photophysical properties in both solution and solid-state of 4a–g were investigated, and theoretical calculations at the DFT and TD-DFT levels were used to interpret the absorption and emission observations.

## Results and discussion

### Synthesis

A family of 7-substituted 2-methylpyrazolo[1,5-*a*]pyrimidines 4a–g was synthesized by a two-step synthesis sequence starting from the appropriate methyl ketone 1a–g. Compounds 4a–g were synthesized in an overall yield of 67–93% by some variations of protocols previously reported in our lab.^12,13^ For example, we started our work by preparing the precursor β-enaminones 2a–b and 2d–g in high yields (83–97%) *via* a solvent-free condensation reaction of the respective methyl ketones (1a–b and 1d–g, 1.0 mmol) with an excess of *N*,*N*-dimethylformamide-dimethylacetal (DMF-DMA, 1.5 mmol) under MW irradiation (MWI) at 160 °C for 15 min. However, the β-enaminone–coumarin derivative 2c could only be obtained (in 87% yield) under reflux for 6 h from an equimolar mixture (1 mmol) of 3-acetyl-2*H*-chromen-2-one (1c) and DMF-DMA in 1,4-dioxane (Scheme 1).^26^

Subsequently, we examined the solvent-free reaction between an equimolar mixture (0.5 mmol) of the appropriate β-enaminone 2 and 3-methyl-1*H*-pyrazol-5-amine (3) under MWI at 180 °C.^12^ Importantly the 7-aryl-3-methylpyrazolo[1,5-*a*]pyrimidines (4a, 4b, 4d, 4e, and 4g) were obtained in 88–96% yield, while the novel hybrid pyrazolo[1,5-*a*]pyrimidines–coumarin systems 4c and 4f were obtained (in 80–87% yield) only under reflux for 3 h in acetic acid (1.0 mL). It is likely that the coumarin derivatives (2c, 2g, 4c, and 4f) decomposed under MW conditions at 180 °C (Scheme 1).

### Green chemistry performance

Since fluorophores 4a–g can be recognized as fine chemicals, for practical applications these compounds are required to fulfill some environmental issues related to their production. Therefore, a univariate green metrics analysis based on a common criterion such as reaction mass efficiency (RME) was performed^27^ (Tables 1 and S1–S7†). The RME values found for this family of 7-substituted 2-methylpyrazolo[1,5-*a*]pyrimidines 4a–g were in the range of 40–53%. The high overall yield and the absence of catalysts explained the good RME performance for the synthesis of 4a–g. Remarkably, the sub-products generated during this synthesis were methanol, dimethylamine and water, useful solvents that can be recovered for use in other chemical processes. To compare the good merits found in these probes, the same calculations were performed for three BODIPY-based fluorophores, a widely used compound in materials science^28^ (Tables 1 and S8–S10†). The overall yield and the RME were better for PPs 4a–g than for BODIPY derivatives.

**Fig. 1:**
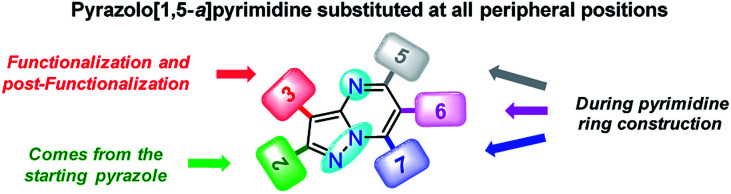
Structural versatility of pyrazolo[1,5-*a*]pyrimidine-based N-heterocycles.

With the use of the MW-assisted technology in almost all procedures, a tool known for its benefits such as easy handling, rapid and solvent-free synthesis, the generation of fewer sub-products,^29^ and maximizing the use of raw materials indicate that the synthetic approach for these fluorophores is in agreement with the main principle of the green chemistry, prevention.^30^ The cost per gram of the raw materials of PPs 4a–e is just a fraction of that calculated for the fluorophores BODIPY-1–BODIPY-3 (Table 1). Meanwhile, the combination of two fluorophores in the hybrid systems 4f and 4g noticeably increases the cost of the product as a result of the need to employ expensive raw materials; however, the preliminary cost per gram of these compounds is better than that of BODIPY-1 and BODIPY-3. The pyrazolo[1,5-*a*]pyrimidine core can be easily modified by simple aromatic substitution reactions such as nitration, halogenation and formylation.^7,12,13^ As a result, suitable functional groups can be incorporated at positions 2 and 5–7 during the fused-ring construction and at position 3 *via* functionalization and post-functionalization strategies (Fig. 1), demonstrating the capability of this fluorophore for structural diversity. Along this direction, we recently demonstrated the improvement in the photophysical properties with the incorporation of a conjugated substituent at position 2.^31^

**Table 1:** Reaction mass efficiency for fluorophores 4a-g and BODIPY derivatives^a^

Compound	Overall yield (%)	RME^a^ (%)	Cost^b^ per g (USD)
4a	85	43	2.7
4b	76	21	1.9
4c	67	42	6.1
4d	93	48	1.9
4e	90	51	1.7
4f	72	41	95.9
4g	77	42	65.7
BODIPY-1 (ref. 32)	19	1.3	98.0
BODIPY-2 (ref. 33 and 34)	50	17.9	35.8
BODIPY-3 (ref. 35)	38	4.4	162.6

a As the starting point we used commercially available raw materials. All calculations were made as the reaction pathway started from 1.0 mmol. Solvent and silica gel used for chromatographic separations were not taken into account.

b For cost per gram calculations, the Sigma-Aldrich prices of the on-line catalog were consulted on August 25, 2020.

### UV–vis and emission spectra

**Fig. 2:**
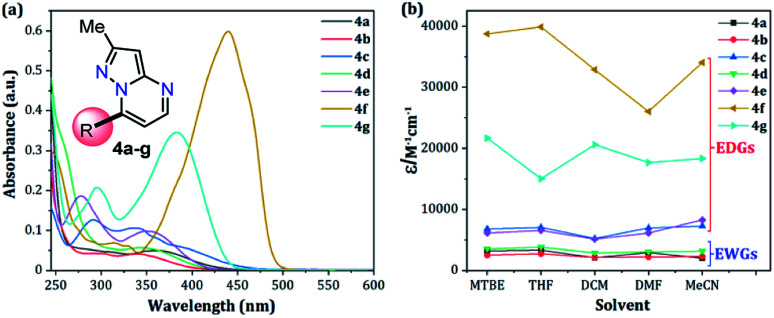
(a) Absorption spectra of compounds 4a–g in THF. (b) Plot of the absorption coefficients of probes 4a–g*versus* solvent (1 × 10^−5^ M) at 20 °C.

**Fig. 3:**
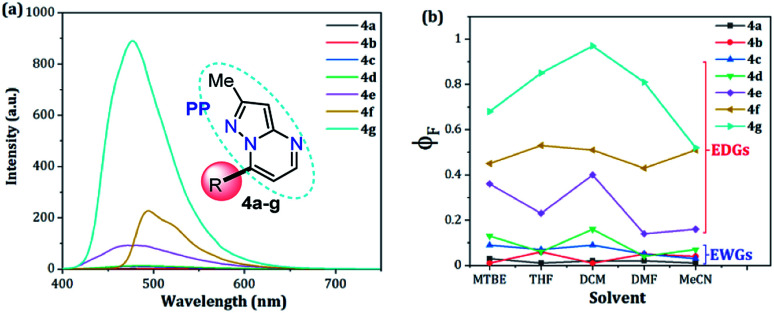
(a) Emission spectra of compounds 4a–g in THF. (b) A plot of the quantum yields of probes 4a–g*versus* solvent (1 × 10^−5^ M) at 20 °C.

The UV-vis and fluorescence spectra of the 7-substituted 3-methylpyrazolo[1,5-*a*]pyrimidines 4a–g were measured in different solvents (Fig. 2 and 3 and Table 2). The absorption and emission spectra of these PPs are highly dependent on the nature of the substituent group at position 7. In general, the absorption spectra of 4a–g showed a main band between 340–440 nm that was previously assigned to an intramolecular charge transfer process.^13,36,37^ Notably, with good EDGs such as 4-anisyl 4e, 7-diethylaminocoumarin-3-yl 4f and 4-diphenylaminophenyl 4g, the absorption coefficient (*ε*) in THF was as high as 6547, 39 867 and 15 008 M^−1^ cm^−1^, respectively. Meanwhile, the presence of EWGs in compounds 4a–c, or neutral groups (NG) such as phenyl in the derivate 4d, decreased the values of *ε* as follows: 4a (3320 M^−1^ cm^−1^), 4b (2727 M^−1^ cm^−1^), 4c (7053 M^−1^ cm^−1^), and 4d (3827 M^−1^ cm^−1^) in the same solvent. In particular, coumarin derivatives 4c and 4f displayed the same differences in the absorption spectra as a result of the π-extended conjugation in the coumarin unit. For example, the absorption coefficient of the intramolecular charge transfer (ICT) band at 440 nm for 4f displayed a much higher *ε* in THF when compared with that of 4d in the same solvent. A broad absorption band with different shoulders was also observed in compound 4c; this behavior can be attributed to a combination of the ICT phenomenon, as well as the π–π* and n–π* transitions of the coumarin and pyrazolo[1,5-*a*]pyrimidine moieties. In all cases, the absorption maximum wavelengths (*λ*_abs_) were almost unchanged, irrespective of the solvent used, while their molar absorption coefficients tended to show a subtle decrease as the solvent polarity increased.

**Table 2:** Photophysical data of fluorophores 4a–g at 20 °C^a^

Entry, R–PP	Solvent	Δ*f*	Abs, nm (*ε*, M^−1^ cm^−1^)	Em^b^, nm (*ϕ*)	Stokes shift, cm^−1^
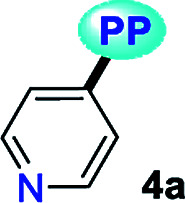	MTBE	0.1413	363 (3187)	489 (**0.03**)	7098
	THF	0.2010	361 (3320)	493 (0.01)	7417
	DCM	0.2221	363 (2073)	506 (0.02)	7785
	DMF	0.2742	360 (2927)	494 (0.02)	7535
	ACN	0.3055	342 (2001)	500 (0.02)	9240
	SS	—	—	492 (0.22)	—
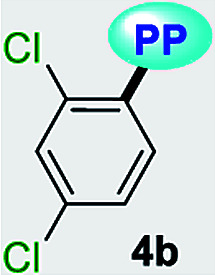	MTBE	0.1413	341 (2500)	479 (**0.09**)	8449
	THF	0.2010	340 (2727)	481 (0.07)	8622
	DCM	0.2221	343 (2120)	486 (**0.09**)	8578
	DMF	0.2742	338 (2213)	490 (0.05)	9178
	ACN	0.3055	355 (2247)	487 (0.03)	7635
	SS	—	—	479 (0.63)	
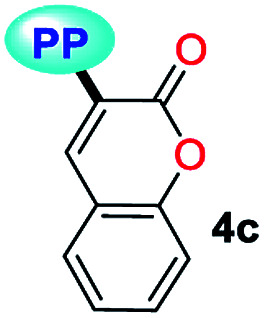	MTBE	0.1413	340 (6787)	523 (**0.07**)	10 291
	THF	0.2010	339 (7053)	535 (**0.06**)	10 807
	DCM	0.2221	339 (5233)	541 (0.01)	11 014
	DMF	0.2742	331 (6953)	503 (0.05)	10 331
	ACN	0.3055	331 (7267)	520 (0.04)	10 981
	SS	—	—	520 (0.01)	—
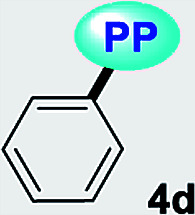	MTBE	0.1413	352 (3520)	486 (**0.13**)	7833
	THF	0.2010	345 (3827)	482 (0.06)	8239
	DCM	0.2221	350 (2849)	478 (**0.16**)	7651
	DMF	0.2742	349 (3040)	490 (0.04)	8245
	ACN	0.3055	345 (3133)	484 (0.07)	8324
	SS	—	—	475 (0.39)	
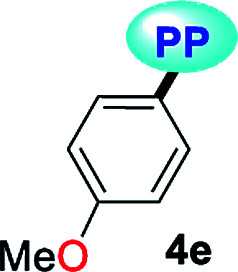	MTBE	0.1413	349 (6127)	476 (**0.36**)	7645
	THF	0.2010	349 (6547)	478 (0.23)	7733
	DCM	0.2221	353 (5128)	479 (**0.40**)	7452
	DMF	0.2742	351 (6120)	478 (0.14)	7570
	ACN	0.3055	345 (8262)	478 (0.16)	8065
	SS	—	—	488 (0.18)	
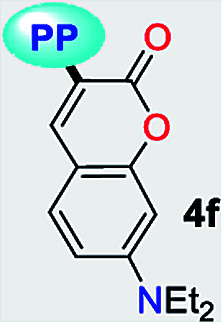	MTBE	0.1413	433 (38 740)	483 (0.45)	2391
	THF	0.2010	440 (39 867)	494 (**0.53**)	2484
	DCM	0.2221	440 (32 867)	496 (**0.51**)	2566
	DMF	0.2742	424 (26 027)	505 (0.43)	3783
	ACN	0.3055	424 (34 033)	501 (0.51)	3625
	SS	—	—	538 (0.08)	
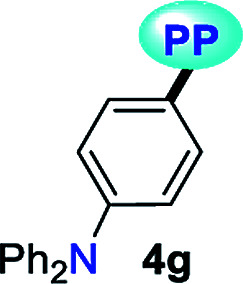	MTBE	0.1413	378 (21 667)	474 (0.68)	5288
	THF	0.2010	387 (15 008)	476 (**0.85**)	4831
	DCM	0.2221	385 (20 593)	488 (**0.97**)	5415
	DMF	0.2742	384 (17 667)	510 (0.81)	6366
	ACN	0.3055	379 (18 313)	512 (0.52)	6854
	SS	—	—	520 (0.13)	

a Data recorded in different solvents (1 × 10^−5^ M) and in the solid-state (SS).

b Relative quantum yield using Prodan as a standard in solution and apparent quantum yield for probes in SS.^38^

When PPs 4a–g were excited at their *λ*_abs_ in an air-equilibrated solution at 20 °C, they exhibited fluorescence bands at around 474–541 nm (Fig. 3a). For these probes, there was also an important correlation between the fluorescence quantum yield (*ϕ*_F_) and the electronic nature of the substituent at position 7 (Fig. 3b). In a solvent of intermediate polarity (*i.e.*, THF), substituents at position 7, known as EDGs, such as anisyl (4e), 7-diethylaminecoumarin-3-yl (4f) or 4-diphenylaminophenyl (4g), the quantum yield values were 0.23, 0.53 and 0.85, respectively. Notably, with EWGs or NG (7-Ph), the *ϕ*_F_ values decreased to 0.01 (4a), 0.07 (4b), 0.06 (4c), and 0.06 (4d) in the same solvent. The fact that the *ϕ*_F_ values in fluorophores 4f and 4g are remarkably high in solvents of different polarity is a notable virtue that can be useful, for example, in monitoring macromolecules dynamics^5^ or labeling pharmacological targets.^3^ Interestingly, these probes displayed good emission intensities even in polar solvents such as ethanol–water 4 : 1 or THF-water mixtures (Fig. S3†), which is a remarkable property for biological applications.

### Solvatofluorochromism

**Fig. 4:**
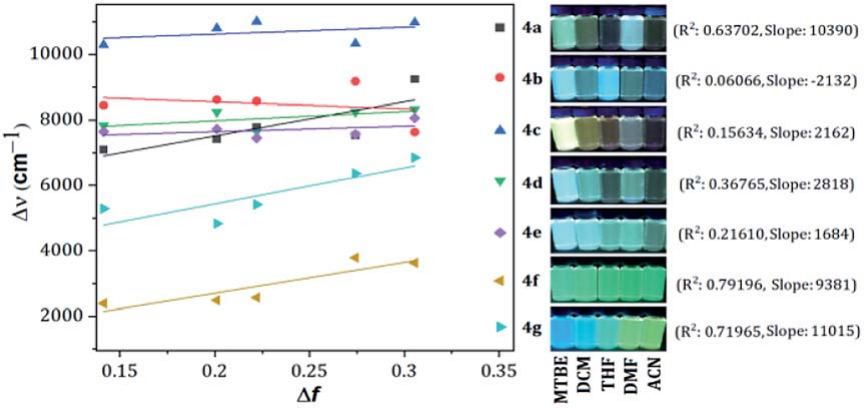
Plots of Δ*ν versus* Δ*f* in different solvents for 4a–g. Photographs were taken using 1.0 × 10^−5^ M solutions of each probe under UV light of 365 nm.

Compounds bearing both EDGs and EWGs as substituents, known as push–pull structures, ensure that after light absorption, the charge is transferred from the donor group to the acceptor, which creates a highly dipolar excited state (DES). The DES interacts with the dipoles of the solvent and thus, the emission shifts to longer wavelengths in more polar solvents.^1,39^ Therefore, the solvatofluorochromic behavior in the emission spectra is a measure of the push–pull properties of a compound. In order to evaluate the solvatochromic features of PPs 4a–g, the relationship between the solvent polarity parameter (Δ*f*) and the Stokes shift (Δ*ν*), according to the Lippert–Mataga equation, was investigated.^40^ From the plots of Δ*ν versus* Δ*f* (Fig. 4), it was found that the slopes of the fitting lines for 4a, 4f and 4g were high, at 10 390, 9381 and 11 015, with acceptable linearity, suggesting that the ICT in these compounds have a larger dipole moment than the ground state due to important charge redistribution. The dipole moment changes (Δ*μ*) in those compounds were calculated to be 10.3, 12.8 and 19.0 D. Interestingly, compound 4a with pyridine as an EWG at position 7 displayed a stronger solvatofluorochromic effect than that observed in 4e bearing an EDG in the same position. This behavior can be explained by the π-amphoteric donor/acceptor property in the pyrazolo[1,5-*a*]pyrimidine core, explicitly a π-excedent–π-deficient fused system. The 7-pyridyl substituent in 4a acts as an EWG and the fused-ring moiety is expected to be the EDG (*i.e.*, a A–A–D molecular system based on pyridine, pyrimidine and pyrazole rings). The results indicated that the structural arrangement in 4a displays a better push–pull system than that observed for fluorophore 4e. Meanwhile, probes 4b–e showed non-solvent polarity dependence in the emission properties because of the absence of strong EDG or EWG substituents, which reduces the charge reorganization in the excited state (Fig. 6).

### Solid-state fluorescence

**Fig. 5:**
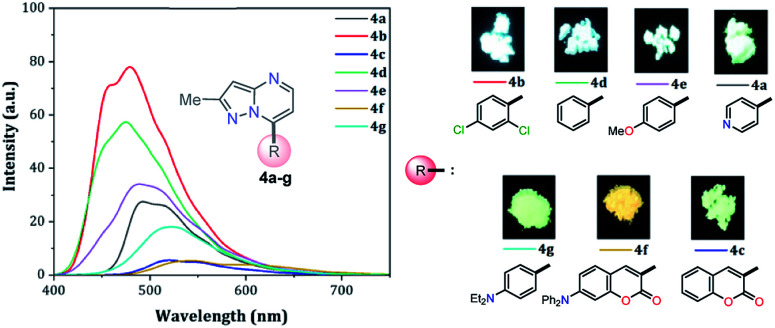
Solid-state emission spectra of compounds 4a–g. Photographs were taken using solid-state samples for each probe under UV light of 365 nm.

Solid-state fluorescence properties of compounds 4a–g were first evaluated upon illumination of each solid sample with UV light of 365 nm using a hand lamp. Fluorophores 4b, 4d, 4a and 4e exhibited a strong blue light emission with quantum yields in solid-state (QY_SS_) of 0.63, 0.39, 0.22 and 0.18, respectively, while other probes were less emissive (QY_SS_: 4g (0.13) > 4f (0.08) > 4c (0.01)) and the fluorescence was red-shifted into the green region of the spectrum (Table 2). Solid-state fluorescence spectra of 4e–g were red-shifted with respect to those in solution, indicating the presence of important intermolecular interactions in the solid-state. Likewise, the emission wavelength of probes 4b, 4d, and 4e are very close to those in solution, suggesting that the fluorescence properties in the solid-state are highly dependent on different structural arrangements due to the nature of the substituent at position 7 (Fig. 5).

**Fig. 6:**
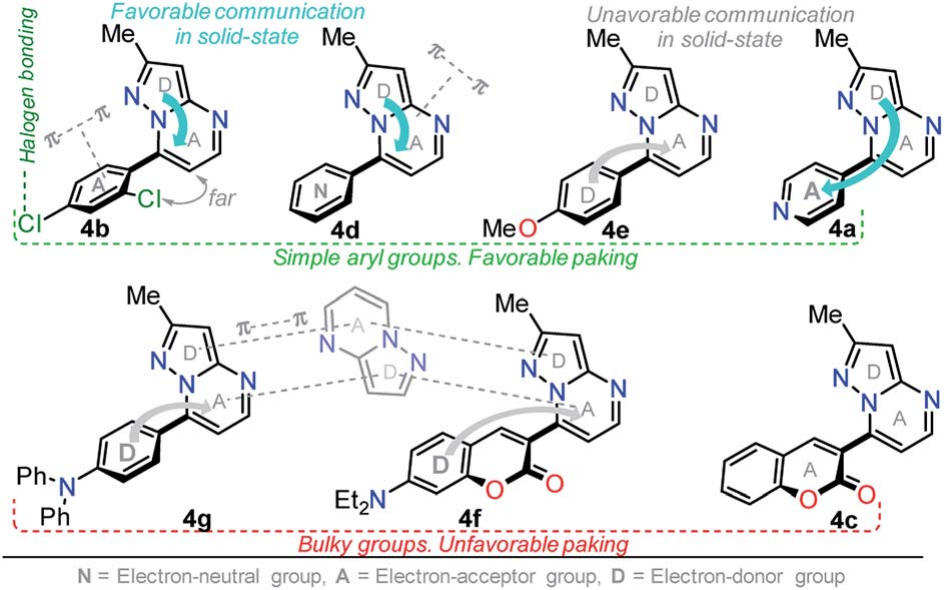
Possible solid-state molecular effects on pyrazolo[1,5-*a*]pyrimidines 4.

It is worth noting that in the donor–acceptor molecular systems, the microcrystalline arrangement allows an intermolecular fluorescence quenching due to the antiparallel organization of the molecules.^31,41,42^ Likewise, in N-heterocyclic compounds that lack strong electrostatic interactions due to the absence of polar functional groups, the van der Waals forces are mainly responsible for their formation in the solid-state and apparently, these forces are improved by the presence of simple aryl groups at the ring periphery. The supramolecular assembly can be promoted by the molecular chain formations having aryl groups or fused heterocyclic moieties in a strategic disposition of π⋯π stacking and C–H⋯π (or C–H⋯N) interactions.^31,43–47^ Some haloaryl substituents play a crucial role in molecular packing and the photophysical properties of their derivatives, such as the 2,4-dichlorophenyl (2,4-Cl_2_Ph) group.^44,45^ In this context and as expected, probes 4c, 4f and 4g showed lower emission intensities with respect to those found in the rest of the series (intensity in 4b > 4d > 4e > 4a), since they bear bulky groups that disfavor both good packing and fluorescence intensity (Fig. 5 and 6).

Notably, the pyrazolo[1,5-*a*]pyrimidine core has been widely studied and various crystal arrangements were reported,^12,13,31,46^ evincing a molecular packing with parallel^46^ or quasi antiparallel^12,13^ dispositions with respect to each other, which depend on the nature of the substituents. However, with strong donor groups at position 7 and without steric effects in other fused ring positions, an utterly antiparallel molecular organization is favored (Fig. 6).^31^ Therefore, the absence of a push–pull structure in 4b, 4g and 4f, avoids the intermolecular quenching in the solid-state,^31,41,42^ allowing high-emission intensities with regard to that observed in derivatives bearing a strong push–pull arrangement (4a-inside of the PP core, 4g and 4f) or bulky substituent groups (4c, 4g and 4f). Finally, the presence of the 2,4-dichlorophenyl substituent in 4b favors its emission intensity as a result of the marked dihedral angle between this group with the heterocyclic core due to the Cl atom at position 2 of this group.^44,45^ This effect could reduce the electronic communication between the two rings^45^ and result in aggregation-induced emission (AIE) phenomenon^47^ (Fig. 5 and 6).

### Probes 4a–g stability

**Fig. 7:**
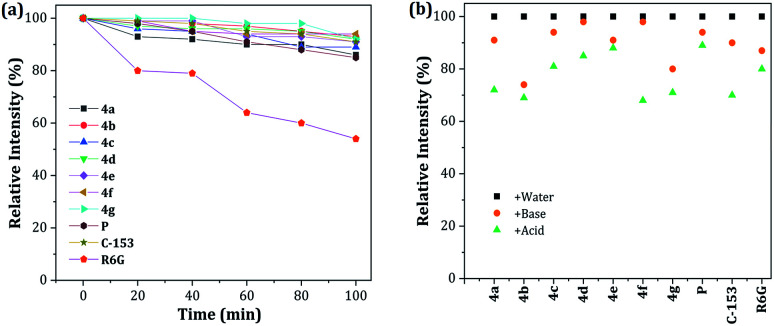
(a) Photostability of 4a–g, P, C-153, and R6G in THF–water 4 : 1. (b) Stability at pH 2 (H_2_SO_4_) and pH 12 (KOH). The concentration of the probes was 1 × 10^−5^ M.

The photobleaching characteristics and stability at extreme pH values of fluorescent compounds are critical to their application as fluorescent materials and bio-probes, respectively. Accordingly, the photostability of dyes 4a–g was investigated and compared with that of prodan (P), coumarin 153 (C-153) and rhodamine 6G (R6G). After continuous excitation at 365 nm with a xenon lamp (4.0 mW) at different times, the normalized fluorescence intensities of dyes 4a–g decreased by 89–94%, measured at their maximum wavelength, which is a very good photobleaching performance when compared with those obtained for the commercial probes (Fig. 7a). Likewise, the stability under exposure to extreme pH (pH 2 with H_2_SO_4_ and pH 12 with KOH and stirring for 1 h at 50 °C; after neutralization, the emission spectra were recorded) was studied and the behavior was followed by the relative fluorescence intensity (Fig. 7b). The results are similar to those found in C-153. Interestingly, acidic conditions have a greater impact on the stability of the pyrazolo[1,5-*a*]pyrimidine-based probes. These observations can be attributed to the high electronic density in different atoms of the fused-pyrazole and the acid–base interactions could be the beginning of the chemical decomposition.

### Computational calculations

To have better insight regarding the molecular structures and electronic properties of fluorophores 4a–g, geometry optimization, analytical frequencies, and excited-state energy calculations were performed at the B3LYP lever^48^ with the Ahlrichs def2-TZVP basis set,^49–51^ as implemented in the ORCA 4.2.0 package.^52,53^ For further computational details, please see the attached ESI.†

**Fig. 8:**
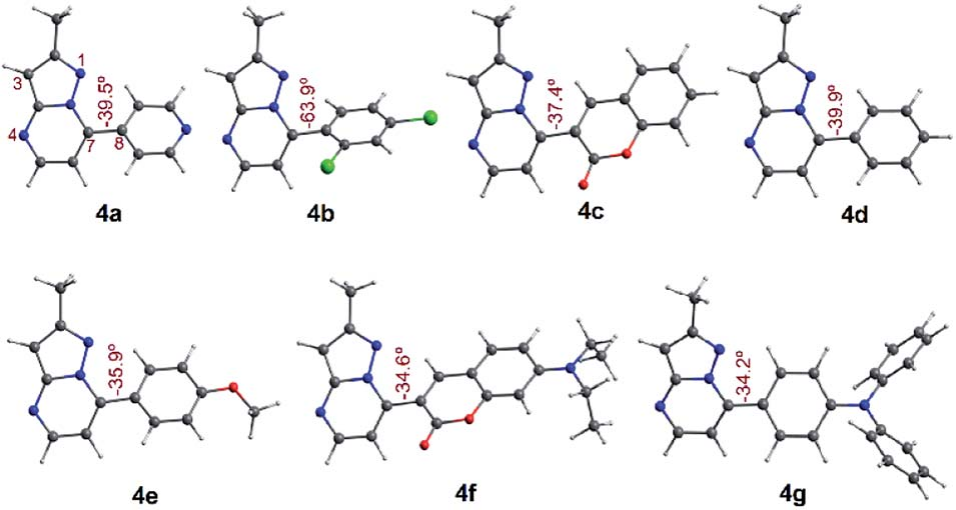
Optimized structures in the singlet ground state of compounds 4a–g in THF. Dihedrals between the PP core and 7-aryl group for fluorophores are shown.

**Fig. 9:**
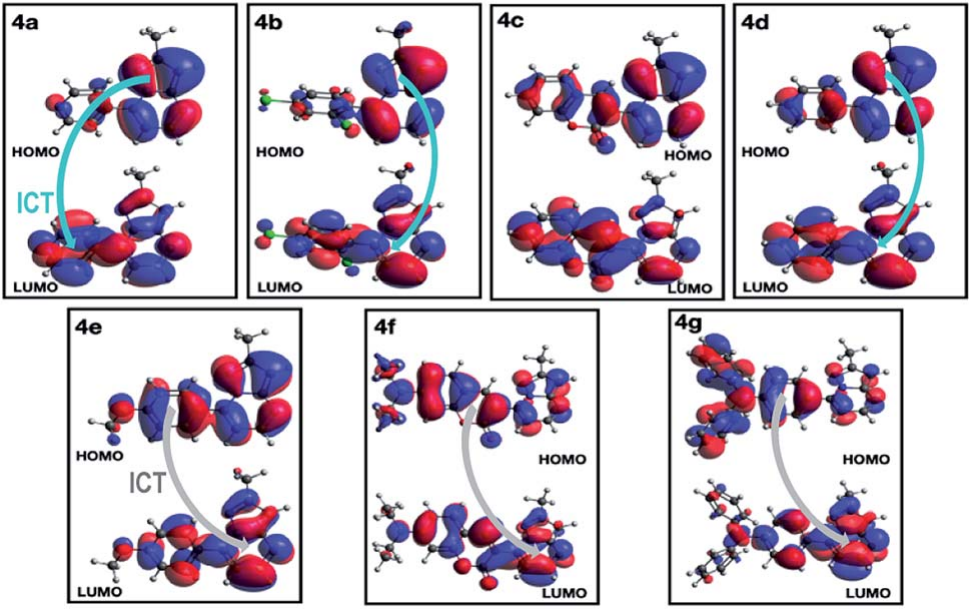
Plot of HOMO and LUMO in the singlet ground state of compounds 4a–g in THF.

All geometry optimization calculations were performed on the framework of the DFT (Fig. 8). This method has been demonstrated to provide reliable results in this kind of system.^54,55^ At the B3LYP level, the ground state of these compounds is a closed-shell singlet (*S* = 0), in which the electronic structure is characterized by frontier molecular orbitals (FMOs) of antibonding π nature (Fig. 9). We performed the geometry optimizations using an implicit solvation model (as described in the ESI†) to evaluate the stability of each probe as a function of the different solvents considered in the experiments (Tables S11–S17†). For the sake of comparison, Table 3 gives the values obtained in THF for the calculated charges on the nucleophilic carbon and nitrogen atoms (C3 and N4 in Fig. 1 and 8), dihedrals and C7–C8 bond lengths connecting the N-heterocyclic core to 7-aryl groups, the HOMO–LUMO gaps, and polarizabilities. The negative charges in C3 and N4 are comparable, ranging from −0.300 to −0.200, which explains the greater reactivity and regioselectivity of these compounds toward electrophilic species.^12,13^

**Table 3:** Mülliken charges in C3 and N4, C7–C8 bond lengths (Å) and the dihedral angle (°), polarizability (Å^3^), and HOMO–LUMO gap (eV), of compounds 4a–g in THF

	4a	4b	4c	4d	4e	4f	4g
Charge N4	−0.263	−0.265	−0.264	−0.270	−0.274	−0.272	−0.275
Charge C3	−0.266	−0.275	−0.272	−0.271	−0.276	−0.296	−0.279
C7–C8 distance	1.473	1.476	1.468	1.471	1.466	1.463	1.465
Dihedral	−39.5	−63.9	−37.4	−39.9	−35.9	−34.6	−34.2
Polarizability	25.13	29.84	34.11	26.12	29.94	48.56	53.31
HOMO–LUMO	4.065	4.332	3.584	4.222	4.145	3.299	3.481

Importantly, N5 may be subject to alkylation reactions to generate pyrimidinium salts, a key group of compounds in organocatalysis^56^ and biochemical applications.^57^ While the C7–C8 distances remain virtually unchanged with the different aryl substituents (1.470 ± 0.007 Å), the dihedrals are disturbed to a larger amplitude (from 34° to 64°), causing the resonance breaking between the aryl group and the pyrazolo[1,5-*a*]pyrimidine core to different extents. The HOMO–LUMO gap also changes as there are variations in the substituents. The calculated gaps are situated in the energy range from 3.3 to 4.3 eV, which is in agreement with the wavelengths of the UV-vis spectra outlined above. The polarizability doubled from 25 Å^3^ as in 4a up to 53 Å^3^ as in 4g, which is in accordance with a greater dipole moment in the excited states of the latter, and the fact that the EWGs at position 7 lead to higher solvatofluorochromisms.

In PPs 4a–g there was a slight decline in the dihedrals, charges, and gaps as the solvent polarity decreased (Tables S11–S17†). Conversely, the polarizability slightly increased with the enhancement of the solvent polarity. As discussed above for the absorption and emission spectra, this behavior reveals that the polarities of the deemed solvents play a minor role in the electronic structure of the studied compounds, as well as in its geometrical arrangement. More interestingly, PPs 4f and 4g showed the opposite behavior, where the polarizability slightly diminished as the solvent polarity increased. In fact, these systems have larger polarizabilities due to their EDGs (as well as the smaller charges, dihedrals and HOMO–LUMO gaps, Table 3), which is in line with the greater fluorescence (*vide infra*). These results can be understood as follows: the smaller the dihedral, the more effective the π-resonance between the heterocyclic moiety and the 7-aryl group, the polarizability increased, and the HOMO–LUMO gap was reduced. Consequently, the respective probes had improved photophysical and electronic properties because of a more favored ICT process.

A deeper analysis of the absorption and emission processes was achieved based on TD-DFT calculations by taking the optimized geometries as a starting point. In this sense, the energies of the five lowest excited singlet states of fluorophores 4a–g in THF were calculated. These energies allowed us to estimate the absorption wavelength of each excited singlet and their respective oscillator strengths *via* the transition dipole moments. As shown in Table 4 and Fig. S16a,† compounds with EDGs (4e–g) have greater oscillator strengths and higher absorbances. The highest oscillator strengths of compounds 4a–b and d–g, are associated with the first singlet excited state, whereas for 4c, this strength matches the third singlet excited state. Their transition energies have a mean value of 3.41 ± 0.32 kcal × mol^−1^, in harmony with absorptions in the UV-vis region. The wavelengths linked to the absorption towards the cited excited singlets are well aligned with our experimental results in the same solvent (Table 2). In almost all PPs studied, absorptions are dominated by HOMO → LUMO electronic transitions (Table 4), though in 4c, it is mainly of the HOMO-1 → LUMO type. In PPs 4a–d, the HOMO mainly has the π-nature on the PP ring, while the LUMO is largely of π-character on the aryl group (Fig. 9, top). These observations are consistent with the fact that the absorption process is mainly associated with electron transfer from the PP ring to the adjoining aryl group, that is, an ICT to the EWGs and NG. Moreover, since the HOMO and LUMO in 4e–g are largely of π-nature on the aryl and PP rings, respectively, the absorption process is reversed (Fig. 9, bottom). Thus, the charge transfer goes from the EDGs to the PP ring. Ultimately, the absorption and fluorescence spectra of PPs 4a–g were predicted (Fig. S16†), including the all-vibronic transitions, based on the excited singlet of each probe. As expected, the estimated spectral behavior agrees with the experimental data (see ESI† for more details).

**Table 4:** Dominant electronic transitions to the excited state of interest for 4a–g in THF based on TD-DFT calculations. Energies are in eV and wavelengths (*λ*) in nm

Probe	Excited state	Energy	*λ*_cal_/*λ*_exp_	Oscillator strength	Dominant electronic transition
4a	1^1^A	3.31	374.3/361	0.10	95% HOMO → LUMO
4b	1^1^A	3.65	340.0/340	0.09	95% HOMO → LUMO
4c	3^1^A	3.82	324.4/339	0.19	79% HOMO-1→LUMO
4d	1^1^A	3.53	351.7/345	0.13	94% HOMO → LUMO
4e	1^1^A	3.63	342.0/349	0.27	91% HOMO → LUMO
4f	1^1^A	3.11	398.4/440	0.88	84% HOMO → LUMO

**Fig. 10:**
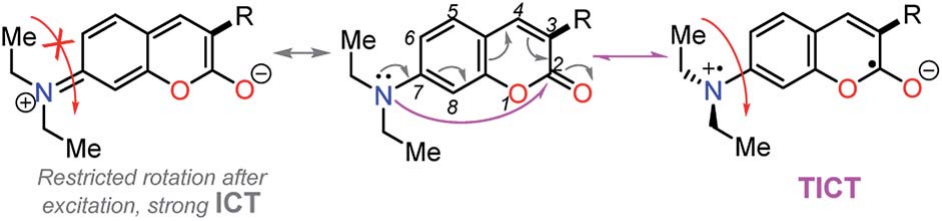
Resonant structures of 7-diethylaminocoumarins in the excited state.

Importantly, compound 4f (7-diethylaminocoumarin-3-yl derivative) displayed lower emission intensity and quantum yield as compared to 4g in optical experiments, which was not reflected in the computational estimations. Likewise, the simple coumarin–PP derivative 4c offered both experimental and calculated photophysical results in lower limits (Fig. 4–6, 8 and S16†). These findings are possibly due to the high sensitivity of the coumarin derivatives to the microenvironments (*e.g.*, solvent properties and solid-state aggregation), steric effects (*e.g.*, groups near the D–A junction and irregular packing) and because 4c does not possess strong EDGs on the coumarin ring, such as the diethylamino (Et_2_N) group at 4f.^58^ In fact, this group offers resonant structures for the two geometries of the 7-Et_2_N-coumarin derivatives in the excited state, from (a) a planar emissive ICT excited state, to (b) a nonfluorescent twisted ICT state (TICT), shown in Fig. 10.^58,59^

## Conclusions

To sum up, we have synthesized a family of fluorescent pyrazolo[1,5-*a*]pyrimidines 4a–g bearing substituent groups of different electronic natures. The photophysical properties of 4a–g in both solution and solid-state were studied and the experimental results were interpreted by means of TD-DFT calculations. From the synthetic point of view, the RME values calculated for compounds were in the range of 40–53%, a notable performance as compared to a widely used fluorophore such as BODIPY (RME: 1.31–17.9%). Consequently, the simple pyrazolo[1,5-*a*]pyrimidine derivatives are raw materials with low-cost, which are better eco-friendly alternatives for developing luminescent compounds. The synthesis is mainly MW-assisted and can be done under solvent-free conditions. Regarding the optical properties of 4a–g, these are highly dependent on the substituent nature, in which EDGs such as anisyl, diethylaminocoumarin and triphenylamine improve both *ε* and ϕ_F_. Meanwhile, the solvatofluorochromic studies showed the amphoteric behavior of PPs and the Lippert–Mataga equation allowed the calculation of dipole moment changes when going from the ground to the excited state (Δ*μ*) for probes 4a (10.3 D), 4f (12.8 D) and 4g (19.0 D). Remarkable solid-state emission intensities were achieved in some compounds with low capability for the ICT phenomenon (QY_SS_ up to 63%). Moreover, these PPs displayed properties comparable with those reported for commercial probes such as Prodan,^40^ Coumarin-153 (ref. 60) and Rhodamine 6G,^61^ and the good photo- and acid–base exposure stability make them attractive alternatives for real applications.

Computational calculations were performed in order to describe the changes in the electronic structure associated with the absorption and emission processes in 4a–g. Geometry optimization calculations on the singlet ground state indicated that the dihedral centered on C7–C8 controls the polarizability and HOMO–LUMO gap of compounds and hence, its respective absorption and fluorescence rates. The smaller the dihedral, the shorter the HOMO–LUMO gap and thus, the ICT phenomena in absorption and emission spectroscopy for the probes are favored. Moreover, the electronic transition analysis of the lowest excited singlet states in probes, revealed that the absorbance experiments at low energy, are governed by a HOMO → LUMO electron transition, while this transition is reversed for emission experiments. The electronic structure analysis based on the FMO nature indicates that EDGs favor large absorption and emission intensities as a result of the ICT process to and from the fused heterocyclic moiety, respectively. When EWGs are used, these intensities remain low, which is in line with the experimental results. Ultimately, both experimental and theoretical results of the coumarin derivatives 4c and 4f provided evidence that the electronic properties of these compounds are governed by different complex phenomena.

## Experimental section

### General procedures

#### General procedure for the synthesis of β-enaminones 2a–g

A 10.0 mL sealable (Teflon screw cap) oven-dried tubular reaction vessel was charged with 1.0 mmol of the appropriate methyl ketone (1a, 1b, 1d, 1e, 1f, or 1g) and 1.5 mmol of DMF-DMA. The resulting mixture was irradiated with MW at 160 °C (180 W) and maintained at this temperature for 15 min in a sealed tube containing a Teflon-coated magnetic stir bar. The resulting reaction mixture was cooled to 55 °C by airflow and the excess of DMF-DMA was removed under reduced pressure, yielding the respective crude β-enaminones (2a, 2b and 2d–g) *via* this protocol previously reported in our lab.^12^ Importantly, β-enaminone 2c was obtained under reflux for 6 h from an equimolar mixture (1 mmol) of 3-acetyl-2*H*-chromen-2-one (1c) and DMF-DMA, according the procedure reported by El-Taweel and Elnagdi;^62^ however, in this case, we used 1,4-dioxane (5.0 mL) as a solvent instead of the xylene used by those authors. Later, the 1,4-dioxane was removed under reduced pressure, yielding the crude product 2c. Finally, all the crude β-enaminones were purified by flash chromatography on silica gel (eluent: CH_2_Cl_2_) to afford the pure products 2d–g.

#### General procedure for the synthesis of pyrazolo[1,5-*a*]pyrimidines 4a–g

A 10.0 mL sealable (Teflon screw cap) oven-dried tubular reaction vessel was charged with an equimolar mixture (0.5 mmol) of the respective β-enaminone (2a, 2b, 2d, 2e, or 2g) and 3-methyl-1*H*-pyrazol-5-amine (3, 49 mg). The resulting mixture was irradiated with MW at 180 °C (200 W) and maintained at this temperature for 2 min in a sealed tube containing a Teflon-coated magnetic stir bar. The resulting reaction mixture was cooled to 55 °C by airflow and the precipitated product formed upon the addition of cold EtOH/H_2_O (1 : 1, 1.0 mL) was filtered off, washed and dried to give the corresponding pure product (4a, 4b, 4d, 4e, and 4g) by this protocol previously reported in our lab.^12^ Meanwhile, fluorophores 4c and 4f were obtained under reflux in acetic acid (1.0 mL) for 3 h, starting from β-enaminone 2c and 2f, respectively. Subsequently, the resulting reaction mixture was concentrated under reduced pressure and the residue was recrystallized from ethanol.

#### Comment

Structures of β-enaminones 2a–g and 7-substituted 2-methylpyrazolo[1,5-*a*]pyrimidines 4a–g were determined *via* NMR measurements and HRMS analysis (see Fig. S4–S15 in ESI†). See the ESI† characterization data and experimental procedure for all synthesized compounds, as well as optical properties (eqn S1 and S2 and Fig. S1–S3†), green metrics (eqn S3, Schemes S3–S5, and Tables S1–S10†), and computational details (Tables S11–S17†) of fluorophores 4a–g.

## Abbreviations

AIE: Aggregation-induced emission
BODIPY: 4,4-Difluoro-4-bora-3a,4a-diaza-*s*-indacene
DES: Dipolar excited state
DMF-DMA: *N*,*N*-Dimethylformamide-dimethylacetal
EDGs: Electron-donating groups
EWGs: Electron-withdrawing groups
ESIPT: Excited state intramolecular proton transfer
FMOs: Frontier molecular orbitals
HOMO: Highest occupied molecular orbital
ICT: Intramolecular charge transfer
LUMO: Lowest unoccupied molecular orbital
MWI: Microwave irradiation
PPs: Pyrazolo[1,5-*a*]pyrimidines
QY_SS_: Quantum yield in solid-state
RME: Reaction mass efficiency
TD-DFT: Time-dependent density functional theory
TICT: Twisted intramolecular charge transfer

## Conflicts of interest

The authors declare no competing financial interest.

## Supplementary Material

RA-010-D0RA07716J-s001

## References

[cit1] Klymchenko A. S. (2017). Solvatochromic and fluorogenic dyes as environment-sensitive probes: design and biological applications. Acc. Chem. Res..

[cit2] Tigreros A., Portilla J. (2020). Recent progress in chemosensors based on pyrazole derivatives. RSC Adv..

[cit3] Nakayama A., Otani A., Inokuma T., Tsuji D., Mukaiyama H., Nakayama A., Itoh K., Otaka A., Tanino K., Namba K. (2020). Development of a 1,3a,6a-triazapentalene derivative as a compact and thiol-specific fluorescent labeling reagent. Commun. Chem..

[cit4] Zhang X., Pan T., Zhang J., Zhang L., Liu S., Xie W. (2019). Color-tunable, spectra-stable flexible white top-emitting organic light-emitting devices based on alternating current driven and dual-microcavity technology. ACS Photonics.

[cit5] Cheng Z., Kuru E., Sachdeva A., Vendrell M. (2020). Fluorescent amino acids as versatile building blocks for chemical biology. Nat. Rev. Chem..

[cit6] Cao D., Liu Z., Verwilst P., Koo S., Jangjili P., Kim J. S., Lin W. (2019). Coumarin-Based Small-Molecule Fluorescent Chemosensors. Chem. Rev..

[cit7] Castillo J.-C., Portilla J. (2018). Recent advances in the synthesis of new pyrazole derivatives. Targets Heterocycl. Syst..

[cit8] Soh N., Ueda T. (2011). Perylene bisimide as a versatile fluorescent tool for environmental and biological analysis: a review. Talanta.

[cit9] Lu H., Mack J., Nyokong T., Kobayashi N., Shen Z. (2016). Optically active BODIPYs. Coord. Chem. Rev..

[cit10] Shindy H. A. (2017). Fundamentals in the chemistry of cyanine dyes: a review. Dyes Pigm..

[cit11] Zhang Q., Wong K. M.-C. (2020). Photophysical, ion-sensing and biological properties of rhodamine-containing transition metal complexes. Coord. Chem. Rev..

[cit12] Castillo J.-C., Rosero H.-A., Portilla J. (2017). Simple access toward 3-halo- and 3-nitro-pyrazolo[1,5-*a*]pyrimidines through a one-pot sequence. RSC Adv..

[cit13] Castillo J.-C., Tigreros A., Portilla J. (2018). 3-Formylpyrazolo[1,5-*a*]pyrimidines as key intermediates for the preparation of functional fluorophores. J. Org. Chem..

[cit14] Bedford R. B., Durrant S. J., Montgomery M. (2015). Catalyst-switchable regiocontrol in the direct arylation of remote C–H groups in pyrazolo[1,5-*a*]pyrimidines. Angew. Chem., Int. Ed..

[cit15] Yang X.-Z., Sun R., Guo X., Wei X.-R., Gao J., Xu Y.-J., Ge J.-F. (2020). The application of bioactive pyrazolopyrimidine unit for the construction of fluorescent biomarkers. Dyes Pigm..

[cit16] Cherukupalli S., Karpoormath R., Chandrasekaran B., Hampannavar G. A., Thapliyal N., Palakollu V. N. (2017). An insight on synthetic and medicinal aspects of pyrazolo[1,5-*a*]pyrimidine scaffold. Eur. J. Med. Chem..

[cit17] Saikia P., Gogoi S., Boruah R. C. (2015). Carbon–carbon bond cleavage reaction: synthesis of multisubstituted pyrazolo[1,5-*a*]pyrimidines. J. Org. Chem..

[cit18] Kumar P. M., Kumar K. S., Mohakhud P. K., Mukkanti K., Kapavarapu R., Parsa K. V. L., Pal M. (2012). Construction of a six-membered fused N-heterocyclic ring *via* a new 3-component reaction: synthesis of (pyrazolo)pyrimidines/pyridines. Chem. Commun..

[cit19] Sun J., Qiu J.-K., Jiang B., Hao W.-J., Guo C., Tu S.-J. (2016). I2-Catalyzed multicomponent reactions for accessing densely functionalized pyrazolo[1,5-*a*]pyrimidines and Their Disulphenylated Derivatives. J. Org. Chem..

[cit20] Hoang G. L., Streit A. D., Ellman J. A. (2018). Three-component coupling of aldehydes, aminopyrazoles, and sulfoxonium ylides *via* rhodium(iii)-catalyzed imidoyl C–H activation: synthesis of pyrazolo[1,5-*a*]pyrimidines. J. Org. Chem..

[cit21] Castillo J.-C., Estupiñan D., Nogueras M., Cobo J., Portilla J. (2016). 6-(Aryldiazenyl)pyrazolo[1,5-a]pyrimidines as strategic intermediates for the synthesis of pyrazolo[5,1-*b*]purines. J. Org. Chem..

[cit22] Chrayteh A., Ewels C., Jacquemin D. (2020). Dual fluorescence in strap ESIPT systems: a theoretical study. Phys. Chem. Chem. Phys..

[cit23] Lodowski P., Maślankiewicz M. J., Jaworska M., Żur L., Pisarski W. A. (2018). Electronic spectra and fluorescence of dithiinodiquinoline compounds. an experimental and theoretical study. J. Lumin..

[cit24] Zhang K., Liu J., Zhang Y., Fan J., Wang C.-K., Lin L. (2019). Theoretical study of the mechanism of aggregation-caused quenching in near-infrared thermally activated delayed fluorescence molecules: hydrogen-bond effect. J. Phys. Chem. C.

[cit25] Tachibana R., Kamiya M., Suzuki S., Morokuma K., Nanjo A., Urano Y. (2020). Molecular design strategy of fluorogenic probes based on quantum chemical prediction of intramolecular spirocyclization. Commun. Chem..

[cit26] Al-Zaydi K. M., Borik R. M., Elnagdi M. H. (2003). 2-Arylhydrazonopropanals as building blocks in heterocyclic chemistry: microwave assisted condensation of 2-arylhydrazonopropanals with amines and active methylene reagents. Molecules.

[cit27] Constable D. J. C., Curzons A. D., Cunningham V. L. (2002). Metrics to ‘green’ chemistry—which are the best?. Green Chem..

[cit28] Liu Z., Jiang Z., Yan M., Wang X. (2019). Recent progress of BODIPY dyes with aggregation-induced emission. Front. Chem..

[cit29] Gopi C., Krupamai G., Dhanaraju M. D. (2019). A recent progress in microwave-assisted synthesis of heterocyclic compounds containing nitrogen, sulphur and oxygen. Rev. J. Chem..

[cit30] Marion P., Bernela B., Piccirilli A., Estrine B., Patouillard N., Guilbot J., Jerome F. (2017). Sustainable chemistry: how to produce better and more from less?. Green Chem..

[cit31] Tigreros A., Macías M., Portilla J. (2020). Photophysical and crystallographic study of three integrated pyrazolo[1,5-*a*]pyrimidine–triphenylamine systems. Dyes Pigm..

[cit32] Wang L., Li L., Cao D. (2017). A BODIPY-based dye with red fluorescence in solid state and used as a fluorescent and colorimetric probe for highly selective detection of cyanide. Sens. Actuators, B.

[cit33] Lee C.-H., Yoon H.-J., Shim J.-S., Jang W.-D. (2012). A boradiazaindacene-based turn-on fluorescent probe for cyanide detection in aqueous media. Chem.–Eur. J..

[cit34] Yu Y., Shu T., Yu B., Deng Y., Fu C., Gao Y., Dong C., Ruan Y. (2018). A novel turn-on fluorescent probe for cyanide detection in aqueous media based on a BODIPY–hemicyanine conjugate. Sens. Actuators, B.

[cit35] Sukato R., Sangpetch N., Palaga T., Jantra S., Vchirawongkwin V., Jongwohan C., Sukwattanasinitt M., Wacharasindhu S. (2016). New turn-on fluorescent and colorimetric probe for cyanide detection based on BODIPY-salicylaldehyde and its application in cell imaging. J. Hazard. Mater..

[cit36] Tigreros A., Castillo J.-C., Portilla J. (2020). Cyanide chemosensors based on 3-dicyanovinylpyrazolo[1,5-a]pyrimidines: effects of peripheral 4-anisyl group substitution on the photophysical properties. Talanta.

[cit37] Tigreros A., Rosero H.-A., Castillo J.-C., Portilla J. (2019). Integrated pyrazolo[1,5-*a*]pyrimidine–hemicyanine system as a colorimetric and fluorometric chemosensor for cyanide recognition in water. Talanta.

[cit38] Würth C., Grabolle M., Pauli J., Spieles M., Resch-Genger U. (2013). Relative and absolute determination of fluorescence quantum yields of transparent samples. Nat. Protoc..

[cit39] Cartwright S. J. (2016). Solvatochromic dyes detect the presence of homeopathic potencies. Homeopathy.

[cit40] Tigreros A., Ortiz A., Insuasty B. (2014). Effect of π-conjugated linkage on photophysical properties: acetylene linker as the better connection group for highly solvatochromic probes. Dyes Pigm..

[cit41] V Ershov O., Ievlev M. Y., Belikov M. Y., Naidenova A. I., Maksimova V. N., Tafeenko V. A. (2017). Synthesis, solution and solid-state fluorescence of 2-diethylaminocinchomeronic dinitrile derivatives. RSC Adv..

[cit42] Sonoda Y., Goto M., Tsuzuki S., Tamaoki N. (2006). Fluorescence spectroscopic properties and crystal structure of a series of donor–acceptor diphenylpolyenes. J. Phys. Chem. A.

[cit43] Charris-Molina A., Castillo J.-C., Macías M., Portilla J. (2017). One-Step synthesis of fully functionalized pyrazolo[3,4-*b*]pyridines *via* isobenzofuranone ring opening. J. Org. Chem..

[cit44] Elejalde N.-R., Butassi E., Zacchino S., Macías M.-A., Portilla J. (2019). Intermolecular interaction energies and molecular conformations in N-substituted 4-aryl-2-methylimidazoles with promising *in vitro* antifungal activity. Acta Crystallogr., Sect. B: Struct. Sci., Cryst. Eng. Mater..

[cit45] Castillo J.-C., Tigreros A., Coquerel Y., Rodríguez J., Macías M. A., Portilla J. (2019). Synthesis of pyrrolo[2,3-*c*]isoquinolines *via* the cycloaddition of benzyne with arylideneaminopyrroles: photophysical and crystallographic study. ACS Omega.

[cit46] Portilla J., Estupiñan D., Cobo J., Glidewell C. (2010). 7-Amino-5-methyl-2-phenyl-6-(phenyldiazenyl)pyrazolo[1,5-a]pyrimidine crystallizes with *Z*′ = 2: pseudosymmetry and the formation of complex sheets built from NH… and CH… pi (arene) hydrogen bonds. Acta Crystallogr., Sect. C: Cryst. Struct. Commun..

[cit47] Zhang L., Chen Y., Jiang J. (2016). Solid state fluorescent functionalized-triphenylamine Bodipy detector for HCl vapor with high stability and absolute fluorescent quantum yield. Dyes Pigm..

[cit48] Weigend F. (2006). Accurate Coulomb-fitting basis sets for H to Rn. Phys. Chem. Chem. Phys..

[cit49] Hellweg A., Hättig C., Höfener S., Klopper W. (2007). Optimized accurate auxiliary basis sets for RI-MP2 and RI-CC2 calculations for the atoms Rb to Rn. Theor. Chem. Acc..

[cit50] Neese F. (2012). The ORCA program system. Wiley Interdiscip. Rev.: Comput. Mol. Sci..

[cit51] Neese F. (2018). Software update: the ORCA program system, version 4.0. Wiley Interdiscip. Rev.: Comput. Mol. Sci..

[cit52] Grimme S., Ehrlich S., Goerigk L. (2011). Effect of the damping function in dispersion corrected density functional theory. J. Comput. Chem..

[cit53] Barone V., Cossi M. (1998). Quantum calculation
of molecular energies and energy gradients in solution by a conductor solvent model. J. Phys. Chem. A.

[cit54] Zarate X., Rodriguez-Serrano A., Schott E., Tatchen J. (2020). DFT/MRCI assessment of the excited-state interplay in a coumarin-Schiff Mg^2+^ fluorescent sensor. J. Comput. Chem..

[cit55] Leonard A. A., Mosquera M. A., Jones L. O., Cai Z., Fauvell T. J., Kirschner M. S., Gosztola D. J., Schatz G. C., Schaller R. D., Yu L., Chen L. X. (2020). Photophysical implications of ring fusion, linker length, and twisting angle in a series of perylenediimide–thienoacene dimers. Chem. Sci..

[cit56] Hartman T., Šturala J., Cibulka R. (2015). Two-phase oxidations with aqueous hydrogen peroxide catalyzed by amphiphilic pyridinium and diazinium salts. Adv. Synth. Catal..

[cit57] Sha X.-L., Yang X.-Z., Wei X.-R., Sun R., Xu Y.-J., Ge J.-F. (2020). A mitochondria/lysosome-targeting fluorescence probe based on azonia-cyanine dye and its application in nitroreductase detection. Sens. Actuators, B.

[cit58] Cao D., Liu Z., Verwilst P., Koo S., Jangjili P., Kim J. S., Lin W. (2019). Coumarin-Based Small-Molecule Fluorescent Chemosensors. Chem. Rev..

[cit59] Jones G., Jackson W. R., Choi C., Bergmark W. R. (1985). Solvent effects on emission yield and lifetime for coumarin laser dyes. Requirements for a rotatory decay mechanism. J. Phys. Chem..

[cit60] Lewis J. E., Maroncelli M. (1998). On the (uninteresting) dependence of the absorption and emission transition moments of coumarin 153 on solvent. Chem. Phys. Lett..

[cit61] Zehentbauer F. M., Moretto C., Stephen R., Thevar T., Gilchrist J. R., Pokrajac D., Richard K. L., Kiefer J. (2014). Fluorescence spectroscopy of Rhodamine 6G: concentration and solvent effects. Spectrochim. Acta, Part A.

[cit62] El-Taweel F. M. A. A., Elnagdi M. H. (2001). Studies with enaminones: synthesis of new coumarin-3-yl azoles, coumarin-3-yl azines, coumarin-3-yl azoloazines, coumarin-3-yl pyrone and coumarin-2-yl benzo[*b*]furans. J. Heterocycl. Chem..

