# Supplementary material for: Pyrazolo[1,5-a]pyrimidines-based fluorophores: a comprehensive theoretical-experimental study
Source: RSC Adv. 2020 Oct 29;10(65):39542–52. doi: 10.1039/d0ra07716j (PMC9057447; doi:10.1039/d0ra07716j)
Supplement: RA-010-D0RA07716J-s001 [file RA-010-D0RA07716J-s001.pdf]

Electronic Supplementary Material (ESI) for RSC Advances.
This journal is © The Royal Society of Chemistry 2020

Electronic supplementary information (ESI)

Pyrazolo[1,5-*a*]pyrimidines based fluorophores: A comprehensive theoretical–experimental study

Alexis Tigreros, Sandra-L. Aranzazu, Nestor-F. Bravo, Jhon Zapata-Rivera, and Jaime Portilla*

Contents

1. **Overview of substrates and products numbering**........p. S2

2. **General scheme for synthesis of this research**........p. S3

3. **Experimental procedures and characterization data**........p. S3
   3.1. General information........p. S3
   3.2. General procedures........p. S4
   3.3. Characterization data........p. S4

4. **Photophysical properties of compounds 4a-g**........p. S8
   4.1. Calculations of quantum yields........p. S8
   4.2. The Lippert-Mataga correlation........p. S8
   4.3. Absorption spectra of compounds 4a-g........p. S9
   4.4. Normalized emission spectra of compounds 4a-g........p. S10
   4.5. Emission spectra of compounds 4a-g in aqueous solutions........p. S11

5. **Copies of NMR spectra**........p. S11

6. **HRMS analysis**........p. S18

7. **Green metrics and cost per gram calculations**........p. S24

8. **Computational details**........p. S29

9. **References**........p. S32

1. Overview of substrates and products numbering

Methyl ketones **1a–g**, dimethylformamide-dimethylacetal (DMF-DMA) and 3-methyl-1*H*-pyrazol-5-amine **3**

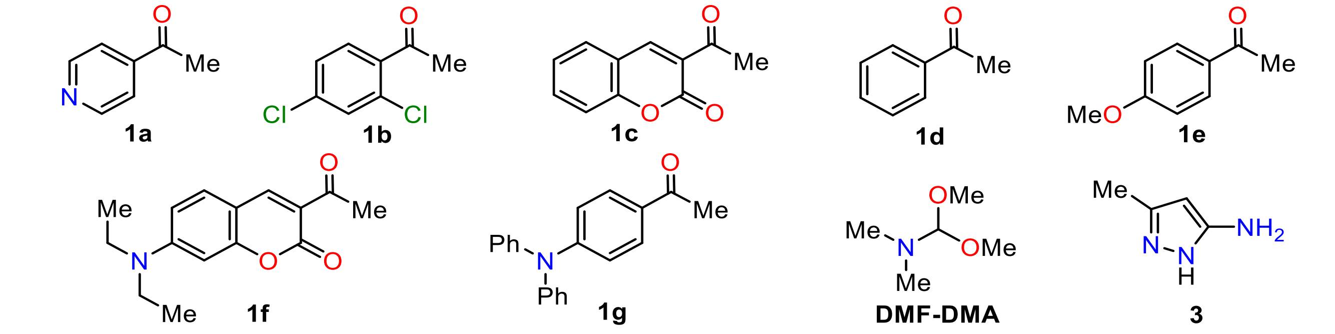

β-Enaminones **2a–g**

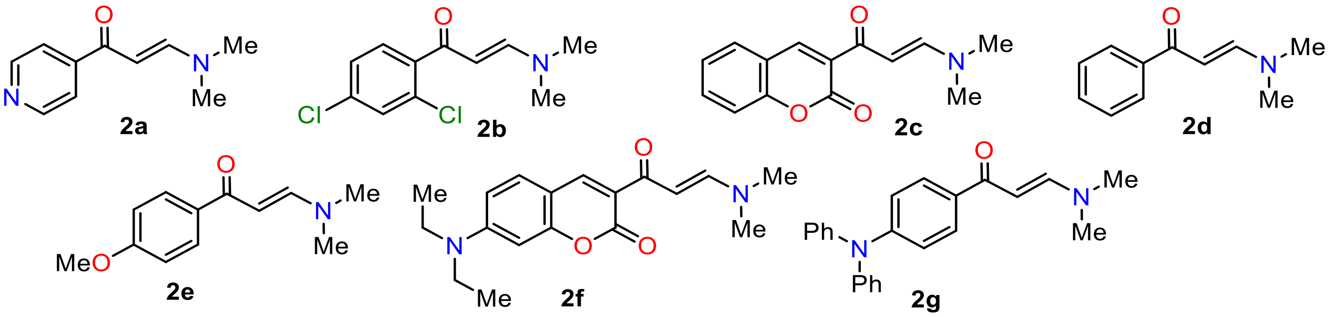

7-Sustituted 2-methylpyrazolo[1,5-*a*]pyrimidines **4a-g**

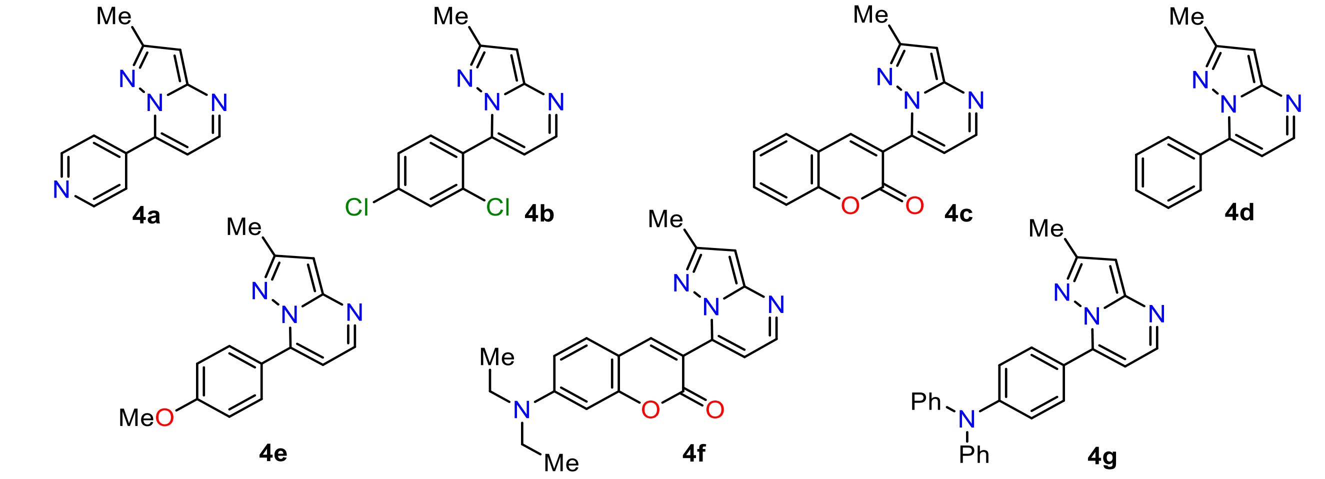

Reference compounds, BODIPYS **1-3**

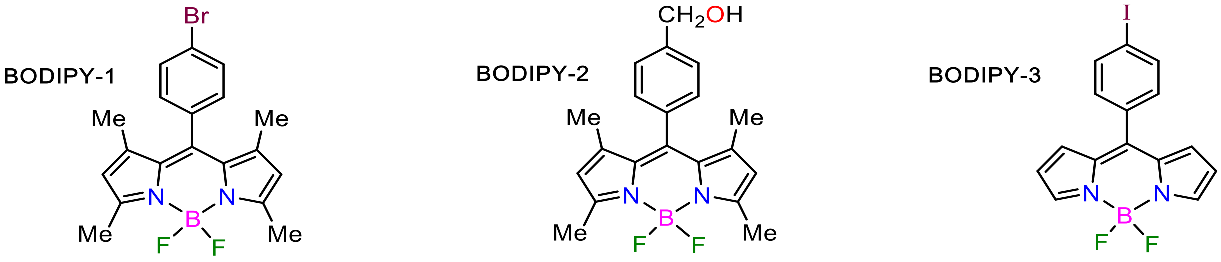

Scheme S1. Structure of all substrates intermediates and products involved in this research

2. General scheme for the synthesis of this research

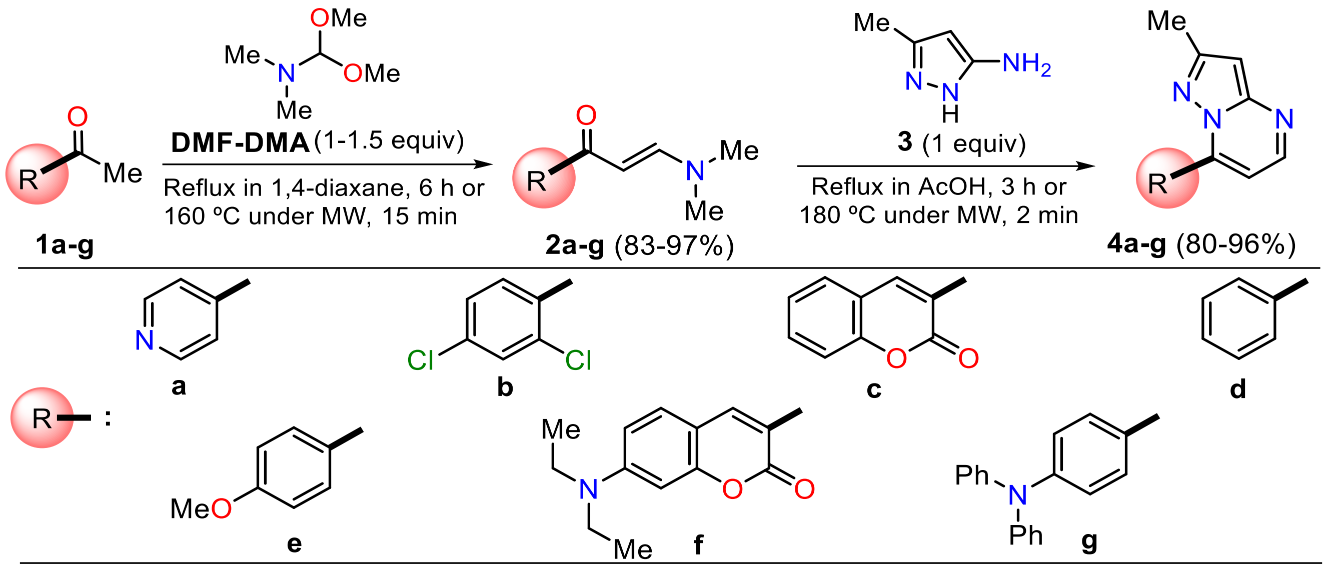

Scheme S2 Synthesis of *β*-enaminones **2a–g** and 7-sustituted 2-methylpyrazolo[1,5-*a*]pyrimidines **4a-g**

3. Experimental procedures and characterization data

3.1. General information

All reagents were purchased from commercial sources and used without further purification unless otherwise noted. All starting materials were weighed and handled in air at room temperature. Progression of reactions and purifications of products were monitored by thin-layer chromatography (TLC) on silica gel (60 F_{254}) by using UV light as visualization agent. Flash chromatography was performed on silica gel (230−400 mesh). All reactions under microwave (MW) irradiation were carried out in a sealed reaction vessel (10.0 mL, max pressure = 300 psi) containing a Teflon-coated stir bar (obtained from CEM) and were performed in a CEM Discover SP focused microwave (ν = 2.45 GHz) reactor equipped with a built-in pressure measurement sensor and a vertically focused IR temperature sensor. Controlled temperature, power, and time settings were used for all reactions. NMR spectra were recorded at 400 MHz (^{1}H) and 101 MHz (^{13}C) at 298 K using tetramethylsilane (0 ppm) as the internal reference and $CDCl_3$ or DMSO-d_6 as solvents. DEPT spectra were used for the assignment of carbon signals. Chemical shifts (δ) are given in parts per million (ppm) and coupling constants (*J*) are given in Hertz (Hz). The following abbreviations are used for multiplicities: s = singlet, d = doublet, t = triplet, and m = multiplet. Melting points were determined using a capillary melting point apparatus and are uncorrected. High-resolution mass spectra (HRMS) were recorded using a Q-TOF spectrometer via electrospray ionization (ESI). The electronic absorption and fluorescence emission spectra were recorded in quartz cuvettes having a path length of 1 cm. UV–vis and fluorescence measurements were performed at room temperature (20 °C). For fluorescence measurements, both the excitation and the emission slit widths were 5 nm.

3.2. General procedures

3.2.1. General procedure for the synthesis of β-enaminones ***2a-g***. A 10.0 mL sealable (Teflon screw cap) oven dried tubular reaction vessel was charged with 1.0 mmol of the appropriate methyl ketone (**1a**, **1b**, **1d**, **1e**, **1f**, or **1g**) and 1.5 mmol of *N,N*-dimethylformamide dimethyl acetal (DMF-DMA). The resulting mixture was irradiated with MW at 160 °C (180 W monitored by an IR temperature sensor) and maintained at this temperature for 15 min in a sealed tube containing a Teflon-coated magnetic stir bar. The resulting reaction mixture was cooled to 55 °C by airflow and the excess of DMF-DMA was removed under reduced pressure yielded the respective crude *β*-enaminones **2a-b** and **2d-g** by this protocol previously reported in our lab.[1] Importantly, *β*-enaminone **2c** was synthesized under reflux for 6 h from an equimolar mixture (1 mmol) of 3-acetyl-2*H*-chromen-2-one (**1c**) and DMF-DMA, according the procedure reported by El-Taweel and Elnagdi;[2] however, in this case we use 1,4-dioxane (5.0 mL) as a solvent instead of xylene. This solvent was removed under reduced pressure yielded the crude product **2c**. Ultimately, all the crude *β*-enaminones were purified by flash chromatography on silica gel (eluent: CH_2Cl_2) to afford the pure products **2a-g**.

3.2.2. General procedure for the synthesis of 7-sustituted 2-methylpyrazolo[1,5-a]pyrimidines ***4a-g***. A 10.0 mL sealable (Teflon screw cap) oven dried tubular reaction vessel was charged with an equimolar mixture (0.5 mmol) of the respective *β*-enaminone (**2a**, **2b**, **2d**, **2e**, or **2g**) and 3-methyl-1*H*-pyrazol-5-amine (**3**, 49 mg). The resulting mixture was irradiated with MW at 180 °C (200 W monitored by an IR temperature sensor) and maintained at this temperature for 2 min in a sealed tube containing a Teflon-coated magnetic stir bar. The resulting reaction mixture was cooled to 55 °C by airflow and the precipitated product formed upon the addition of cold EtOH/H_2O (1:1, 1.0 mL) was filtered off, washed and dried to give the corresponding pure product (**4a**-**b**, **4d**-**e**, and **4g**) by this protocol previously reported in our lab.[1] Meanwhile, fluorophores **4c** and **4f** were obtained under reflux in acetic acid (1.0 mL) for 3 h starting from *β*-enaminone **2c** and **2f**, respectively. Subsequently, the resulting reaction mixture was concentrated under reduced pressure and the residue was recrystallized from ethanol.

3.3. Characterization data

3.3.1. β-Enaninones 2a-g

*1-(E)-3-(Dimethylamino)-1-(pyridin-4-yl)prop-2-en-1-one (**2a**).* Following the general procedure in the reaction with 4-acetylpyridine (**1a**, 121 mg, 1.0 mmol) for 15 min, the compound **2a** was obtained as a brown solid (171 mg, 97%). M.p. 110–111 °C (amorphous) (Lit.[1] 111–113 °C). ^{1}H NMR (400 MHz, $CDCl_3$): δ = 2.89 (s, 3H), 3.13 (s, 3H), 5.60 (d, *J* = 12.0 Hz, 1H), 7.62 (d, *J* = 6.1 Hz, 2H), 7.79 (d, *J* = 12.0 Hz, 1H), 8.63 (d, *J* = 6.0 Hz, 2H) ppm. ^{13}C{^{1}H} NMR (101 MHz, $CDCl_3$): δ = 37.2 (CH_3), 45.1 (CH_3), 91.6 (CH), 121.1 (CH), 147.1 (C), 149.9 (CH), 155.1 (CH), 186.4 (C) ppm. These NMR data matched previously reported data by us.[1]

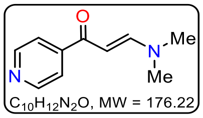

(E)-1-(2,4-dichlorophenyl)-3-(dimethylamino)prop-2-en-1-one ***(2b)***. Following the general procedure in

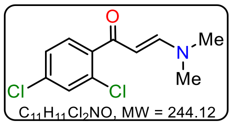

the reaction with 1-(2,4-dichlorophenyl)ethan-1-one (**1b**, 189 mg, 1.0 mmol) for 15 min, the compound **2b** was obtained as a yellow solid (203 mg, 84%). M.p. 73–74 °C (amorphous) (Lit.[3] 71–73 °C). ^{1}H NMR (400 MHz, DMSO-d_6): δ = 2.83 (s, 3H), 3.07 (br s, 3H), 5.20 (d, *J* = 12.4 Hz, 1H), 7.25-7.70 (br m, 4H) ppm. ^{13}C{^{1}H} NMR (101 MHz, DMSO-d_6): δ = 37.5 (CH_3), 45.0 (CH_3), 95.0 (CH), 127.7 (CH), 129.6 (CH), 130.4 (CH), 131.2 (C), 133.9 (C), 134.0 (C), 154.4 (C), 188.3 (C) ppm. HRMS (ESI+): calcd. for $C_{11}H_{12}Cl_2NO^+$ 244.0290 [M + H]$^+$; found 244.0293. The ^{1}H NMR data matched previously reported data.[3]

(E)-1-(3-Coumarinyl)-3-dimethylamino-2-propen-1-one ***(2c)***. Following the general procedure for the

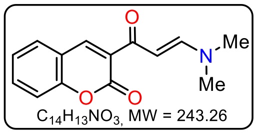

reaction of 3-acetyl-2*H*-chromen-2-one (**1c**, 188 mg, 1 mmol) with DMF–DMA (133 µL, 1 mmol) under reflux in 1,4-dioxane (5.0 mL), the compound **2c** was obtained as an orange solid (212 mg, 87%). Mp. 162–163 °C. (Amorphous) (Lit.[2] 165 °C). ^{1}H NMR (400 MHz, $CDCl_3$ - *d*) *δ* = 2.98 (s, 3H), 3.18 (s, 3H), 6.31 (d, *J* = 12.2 Hz, 1H), 7.27–7.35 (m, 2H), 7.55–7.63 (m, 2H), 7.94 (d, *J* = 12.3 Hz, 1H), 8.59 (s, 1H) ppm. ^{13}C{^{1}H} NMR (101 MHz, $CDCl_3$) *δ*: 37.6 (CH_3), 45.3 (CH_3), 95.3 (CH), 116.4 (CH), 119.1 (C), 124.5 (CH), 126.7 (C), 129.5 (CH), 132.9 (CH), 145.7 (CH), 154.7 (C), 155.1 (CH), 159.9 (C), 182.2 (C) ppm. HRMS (ESI+): Calcd. for $C_{14}H_{14}NO_3^+$ 244.0968 [M+1]$^+$; found 244.0967.

(E)-3-(Dimethylamino)-1-phenylprop-2-en-1-one ***(2d)***. Following the general procedure in the reaction

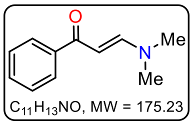

with acetophenone (**1d**, 110 µL, 1 mmol), the compound **2d** was obtained as a yellow solid (170 mg, 97%). Mp 93–95 °C (amorphous) (Lit.[1] 95–96 °C). ^{1}H NMR (400 MHz, $CDCl_3$): δ = 2.92 (s, 3H), 3.11 (s, 3H), 5.71 (d, *J* = 12.4 Hz, 1H), 7.38–7.46 (m, 3H), 7.80 (d, *J* = 12.4 Hz, 1H), 7.89 (d, *J* = 8.2 Hz 2H) ppm. ^{13}C{^{1}H} NMR (101 MHz, $CDCl_3$): δ = 37.2 (CH_3), 44.9 (CH_3), 92.2 (CH), 127.4 (CH), 128.1 (CH), 130.8 (CH), 140.5 (C), 154.2 (CH), 188.6 (C) ppm. These NMR data matched previously reported data by us.[1]

(E)-3-(Dimethylamino)-1-(4-methoxyphenyl)prop-2-en-1-one ***(2e)***. Following the general procedure in the

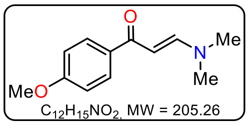

reaction with 4-methoxyacetophenone (**1e**, 138 µL, 1 mmol), the compound **2e** was obtained as a yellow solid (195 mg, 95%). Mp 95–97 °C (amorphous) (Lit.[1] 97 °C). ^{1}H NMR (400 MHz, $CDCl_3$): δ = 2.99 (br s, 6H), 3.82 (s, 3H), 5.68 (d, *J* = 12.3 Hz, 1H), 6.88 (d, *J* = 8.8 Hz, 2H), 7.75 (d, *J* = 12.4 Hz, 1H), 7.88 (d, *J* = 8.8 Hz, 2H) ppm. ^{13}C{^{1}H} NMR (101 MHz, $CDCl_3$): δ = 37.2 (CH_3), 44.9 (CH_3), 55.2 (CH_3), 91.6 (CH), 113.2 (CH), 129.3 (CH), 133.0 (C), 153.7 (CH), 161.8 (C), 187.3 (C) ppm. These NMR data matched previously reported data by us.[1]

(E)-7-(Diethylamino)-3-(3-(dimethylamino)acryloyl)-2H-chromen-2-one ***(2f)***. Following the general

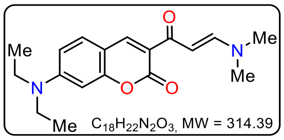

procedure in the reaction with 3-acetyl-7-(diethylamino)-2*H*-chromen-2-one (**1f**, 259 mg, 1 mmol), the compound **2f** was obtained as a brown solid (258 mg, 83%). Mp. 155–157 °C. (Amorphous) ^{1}H NMR (400 MHz, $CDCl_3$) *δ* = 1.18 (t, *J* = 7.2 Hz, 6H), 2.92 (s, 3H), 3.11 (s, 3H), *δ* = 3.39 (m, 4H), 6.41 (m, 2H), 6.56 (d, *J* = 2.4 Hz, 1H), 7.35 (d, *J* = 8.9 Hz,

1H), 7.85 (d, *J* = 12.5 Hz, 1H), 8.50 (s, 1H) ppm. $^{13}C\{^1H\}$ NMR (101 MHz, $CDCl_3$ -*d*) *δ*: 12.4 (CH_3), 37.4 (CH_3), 44.9 (CH_2), 45.0 (CH_3), 95.3 (CH), 96.4 (CH), 108.6 (C), 109.26 (CH), 118.4 (CH), 130.9 (CH), 146.5 (CH), 151.8 (C), 154.2 (CH), 157.7 (C), 161.2 (C), 183.1 (C) ppm. HRMS (ESI+): Calcd. for $C_{18}H_{23}N_2O_3^+$ 315.1703 $[M+1]^+$; found 315.1693.

(E)-3-(Dimethylamino)-1-(4-(diphenylamino)phenyl)prop-2-en-1-one ***(2g)***. Following the general procedure in the reaction with 1-(4-(diphenylamino)phenyl)ethan-1-one (**1g**, 287 mg, 1 mmol), the compound **2g** was obtained as a yellow solid (294 mg, 86%). Mp 133–134 °C (amorphous) (Lit.[4] 134 °C). ^1H NMR (400 MHz, $CDCl_3$) δ: 3.02 (br s, 6H), 5.70 (d, *J* = 12.4 Hz, 1H), 7.01-7.14 (m, 8H), 7.27 (m, 4H), 7.79 (m, 3H) ppm. ^{13}C NMR (101 MHz, $CDCl_3$) δ: 37.5 (CH_3), 44.2 (CH_3), 91.7 (CH), 121.3 (CH), 123.7 (CH), 125.4 (CH), 129.0 (CH), 129.4 (CH), 133.7 (CH), 147.3 (C), 150.6 (C), 153.7 (C), 187.4 (C) ppm. These NMR data matched previously reported data by us.[4]

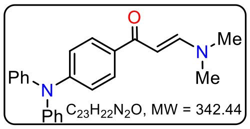

3.3.2. 2-Methylpyrazolo[1,5-a]pyrimidines 4a-g

*2-Methyl-7-(pyridin-4-yl)pyrazolo[1,5-a]pyrimidine (**4a**).* Following the general procedure in the reaction with *β*-enaminone **2a** (88 mg, 0.5 mmol), the product **4a** was obtained as a yellow solid (93 mg, 88%). Mp 176–178 °C (amorphous) (Lit.[1] 177–178 °C). ^1H NMR (400 MHz, $CDCl_3$): δ = 2.52 (s, 3H), 6.60 (s, 1H), 6.85 (d, *J* = 4.4 Hz, 1H), 7.97 (d, *J* = 4.6 Hz, 2H), 8.48 (d, *J* = 4.4 Hz, 1H), 8.83 (d, *J* = 4.6 Hz, 2H) ppm. $^{13}C\{^1H\}$ NMR (101 MHz, $CDCl_3$): δ =14.7 (CH_3), 96.9 (CH), 106.7 (CH), 123.0 (CH), 138.6 (C), 143.1 (C), 148.4 (CH), 150.4 (CH), 150.5 (C), 155.4 (C) ppm. These NMR data matched previously reported data by us.[1]

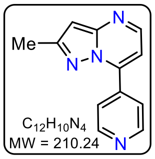

*7-(2,4-Dichlorophenyl)-2-methylpyrazolo[1,5-a]pyrimidine (**4b**).* Following the general procedure in the reaction with *β*-enaminone **2b** (122 mg, 0.5 mmol), the product **4b** was obtained as a yellow solid (127 mg, 91%). Mp 150–151 °C (amorphous). ^1H NMR (400 MHz, $CDCl_3$): δ = 2.48 (s, 3H), 6.58 (s, 1H), 6.73 (d, *J* = 4.3 Hz, 1H), 7.43 (d, *J* = 8.3 Hz, 1H), 7.53 (d, *J* = 8.2 Hz, 1H), 7.60 (d, *J* = 1.9 Hz, 1H), 8.47 (d, *J* = 4.1 Hz, 1H) ppm. $^{13}C\{^1H\}$ NMR (101 MHz, $CDCl_3$): δ =14.8 (CH_3), 96.8 (CH), 108.4 (CH), 127.5 (CH), 129.2 (C), 130.3 (CH), 131.9 (CH), 134.4 (C), 137.1 (C), 142.8 (C), 148.1 (CH), 150.0 (C), 155.4 (C) ppm. HRMS (ESI+): calcd. for $C_{13}H_{10}Cl_2N_3^+$ 278.0246 $[M + H]^+$; found 278.0253.

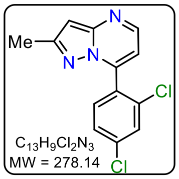

*3-(2-Methylpyrazolo[1,5-a]pyrimidin-7-yl)-2H-chromen-2-one (**4c**).* Following the general procedure under reflux in the reaction with *β*-enaminone **2c** (122 mg, 0.5 mmol), the product **4c** was obtained as a yellow solid (111 mg, 80%). Mp. 209–211 °C (amorphous). °C. ^1H NMR (400 MHz, $CDCl_3$) *δ* = 2.54 (s, 3H), 6.59 (s, 1H), 7.33–7.43 (m, 3H), 7.64–7.69 (m, 2H), 8.48 (d, *J* = 4.4 Hz, 1H), 9.08 (s, 1H) ppm. $^{13}C\{^1H\}$ NMR (101 MHz, $CDCl_3$ -*d*) *δ*: 14.8 (CH_3), 96.9 (CH), 108.5 (CH), 116.8 (CH), 117.9 (C), 118.4 (C), 125.0 (CH), 129.4 (CH), 133.6 (C), 138.9 (CH), 146.2 (CH), 148.3 (CH), 150.5 (C), 154.2 (C), 154.721 (C), 158.4 (C) ppm. HRMS (ESI+): Calcd. for $C_{16}H_{12}N_3O_2^+$ 278.0924 $[M+1]^+$; found 278.0925.

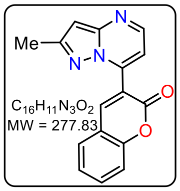

*2-Methyl-7-phenylpyrazolo[1,5-a]pyrimidine (**4d**).* Following the general procedure in the reaction with *β*-enaminone **2d** (88 mg, 0.5 mmol), the product **4d** was obtained as a white solid (100 mg, 96%). Mp: 123–124 °C (amorphous) (Lit.[1] 123 °C). ^{1}H NMR (400 MHz, $CDCl_3$): δ = 2.52 (s, 3H), 6.55 (s, 1H), 6.78 (d, *J* = 4.4 Hz, 1H), 7.53–7.55 (m, 3H), 8.04–8.06 (m, 2H), 8.42 (d, *J* = 4.4 Hz, 1H) ppm. ^{13}C{^{1}H} NMR (101 MHz, $CDCl_3$): δ = 15.2 (CH3), 96.7 (CH), 106.9 (CH), 129.0 (CH), 129.6 (CH), 131.3 (CH), 131.7 (C), 146.5 (C), 149.0 (CH), 151.1 (C), 155.4 (C) ppm. These NMR data matched previously reported data by us.[1]

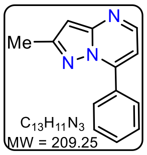

*7-(4-Methoxyphenyl)-2-methylpyrazolo[1,5-a]pyrimidine (**4e**).* Following the general procedure in the reaction with *β*-enaminone **2e** (103 mg, 0.5 mmol), the product **4e** was obtained as a yellow solid (114 mg, 95%). Mp: 126–127 °C (amorphous) (Lit.[1] 128 °C). ^{1}H NMR (400 MHz, $CDCl_3$): δ = 2.53 (s, 3H), 3.90 (s, 3H), 6.53 (s, 1H), 6.76–6.78 (m, 1H), 7.07 (d, *J* = 8.8 Hz, 2H), 8.09 (d, *J* = 8.8 Hz, 2H), 8.39–8.41 (m, 1H) ppm ^{13}C{^{1}H} NMR (101 MHz, $CDCl_3$): δ = 14.8 (CH_3), 55.4 (CH_3), 96.1 (CH), 105.7 (CH), 114.0 (CH), 123.4 (C), 130.9 (CH), 145.9 (C), 148.5 (CH), 150.8 (C), 154.8 (C), 161.7 (C) ppm. HRMS (ESI+): calcd. for $C_{14}H_{14}N_3O^+$ 240.1131 [M + H]$^+$; found 240.1139. These NMR data matched previously reported data by us.[1]

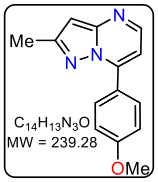

*7-(Diethylamino)-3-(2-methylpyrazolo[1,5-a]pyrimidin-7-yl)-2H-chromen-2-one (**4f**).* Following the general procedure in the reaction with *β*-enaminone **2f** (157 mg, 0.5 mmol), the product **4f** was obtained as an orange solid (152 mg, 87%). Mp: 202–204 °C (amorphous). ^{1}H NMR (400 MHz, $CDCl_3$ - *d*) *δ* = 1.25 (t, *J* = 7.1 Hz, 3H), 2.54 (s, 3H), 3.47 (m, 4H), 6.53 (m, 2H), 6.65 (d, *J* = 8.9 Hz, 1H), 7.44 (m, 2H), 8.43 (d, *J* = 4.6 Hz 1H), 9.14 (s, 1H) ppm. ^{13}C{^{1}H} NMR (101 MHz, $CDCl_3$) *δ*: 12.5 (CH_3), 14.8 (CH_3), 45.1 (CH_2), 96.3 (CH), 96.9 (CH), 107.9 (CH), 107.9 (C), 108.9 (C), 109.5 (CH), 130.9 (CH), 140.4 (C), 146.6 (CH), 148.4 (CH), 150.8 (C), 152.3 (C), 154.1 (C), 157.2 (C), 159.9 (C) ppm. HRMS (ESI$^+$): Calcd. for $C_{20}H_{21}N_4O_2^+$ 349.1659 [M+1]$^+$; found 349.1649.

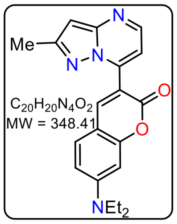

*4-(2-Methylpyrazolo[1,5-a]pyrimidin-7-yl)-N,N-diphenylaniline (**4g**).* Following the general procedure in the reaction with *β*-enaminone **2g** (171 mg, 0.5 mmol), the product **4g** was obtained as a yellow solid (169 mg, 90%). Mp: 162–163 °C (amorphous) (Lit.[4] 161–162 °C). ^{1}H NMR (400 MHz, $CDCl_3$) δ: 2.53 (s, 3H), 6.52 (s, 1H), 6.78 (d, *J* = 4.4 Hz, 1H), 7.23 – 7.06 (m, 8H), 7.32 (t, *J* = 7.8 Hz, 4H), 8.03 (d, *J* = 8.7 Hz, 2H), 8.39 (d, *J* = 4.5 Hz, 1H) ppm. ^{13}C NMR (101 MHz, $CDCl_3$) δ: 15.0 (CH_3), 96.1 (CH), 105.6 (CH), 120.9 (CH), 123.4 (C), 124.4 (CH), 125.9 (CH), 129.7 (CH), 130.5 (CH), 145.9 (C), 147.0 (C), 148.6 (CH), 150.5 (C), 151.1 (C), 154.9 (C) ppm. These NMR data matched previously reported data by us.[4]

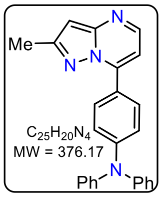

4. Photophysical properties of compounds 4a-g

4.1. Calculations of quantum yields

The photoluminescence quantum yields where determined by using anthracene as a reference standard and according to equation S1.

$$\phi_{f,x} = \phi_{f,st} \frac{F_x}{F_{st}} \frac{A_{st}}{A_x} \frac{n_x^2}{n_{st}^2} \qquad \text{Equation S1.}$$

F is the integral photon flux, A is the absorption factor, n is the refractive index of the solvent and ϕ_f is the quantum yield. The index x denotes the sample, and the index st denotes the standard.[5]

4.2. The Lippert-Mataga correlation.

$$\nu_{Abs} - \nu_{Em} = \frac{2(\mu_e - \mu_g)\Delta f}{hca^3}, \quad \Delta f = \frac{(\varepsilon - 1)}{(2\varepsilon + 1)} - \frac{(n^2 - 1)}{(2n^2 + 1)} \qquad \text{Equation S2.}$$

In equation 1 h is Planck's constant, c is the speed of light, and a is the radius of the cavity in which the fluorophore resides (the onsager radius, from crystal structure), λ_{Abs} and λ_{Em} are the wavenumbers (cm^{-1}) of the absorption and emission, respectively. The term (ε-1)/(2ε-1) accounts for the spectral shifts due to both the reorientation of the solvent dipoles and to the redistribution of the electrons in the solvent molecules and the term (n2 + 1)/(2n + 1) accounts for only the redistribution of electrons. The spectral shifts due to reorientation of the solvent molecules, orientation polarizability (Δf), were obtained from the differences of the latter terms.[6]

4.3. Absorption spectra of compounds 4a-g

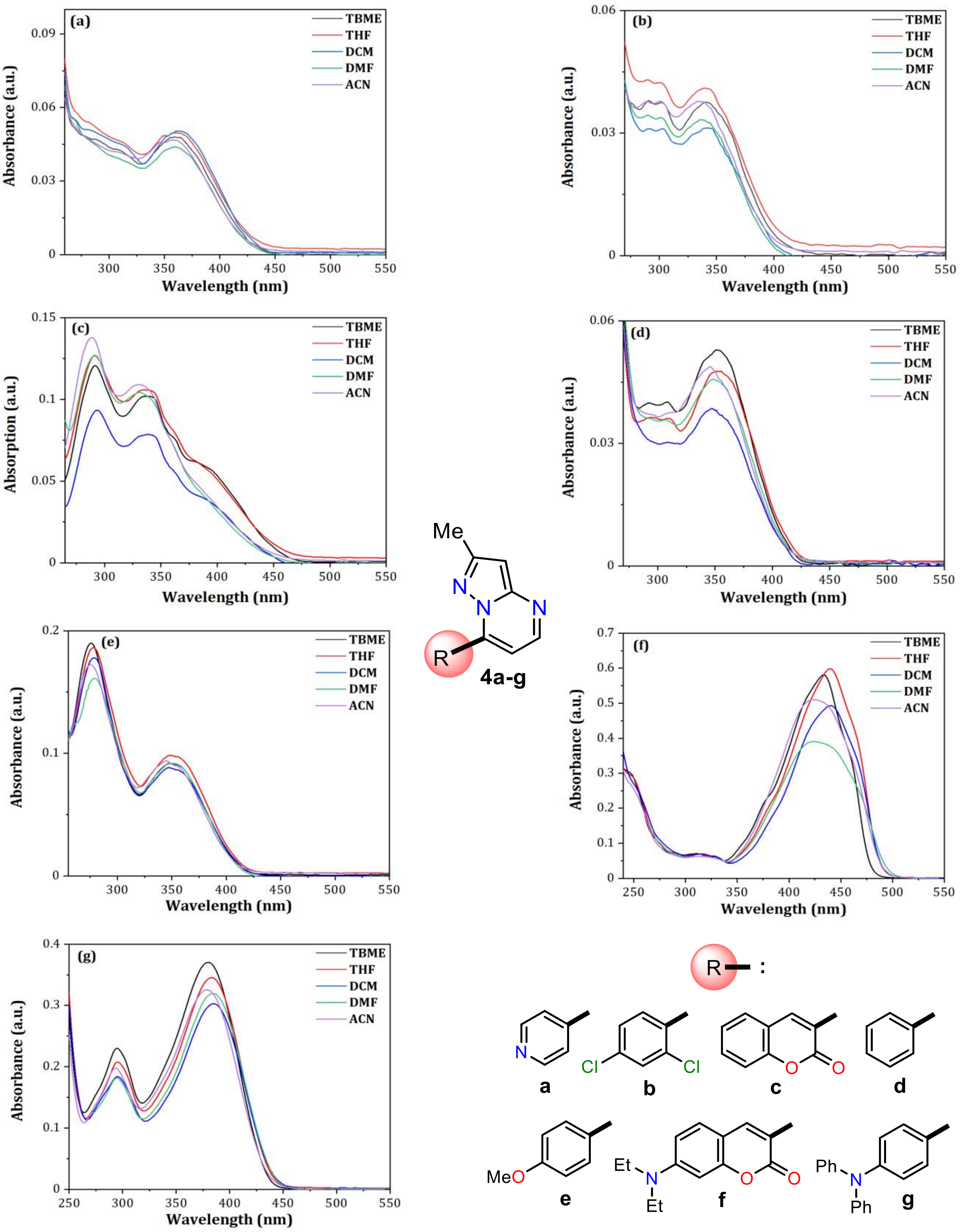

Fig. S1. Absorption spectra in different solvents (1×10^{-5} M and 20 °C) and structures of compounds **4a-g**.

4.4. Emission spectra of compounds 4a-g

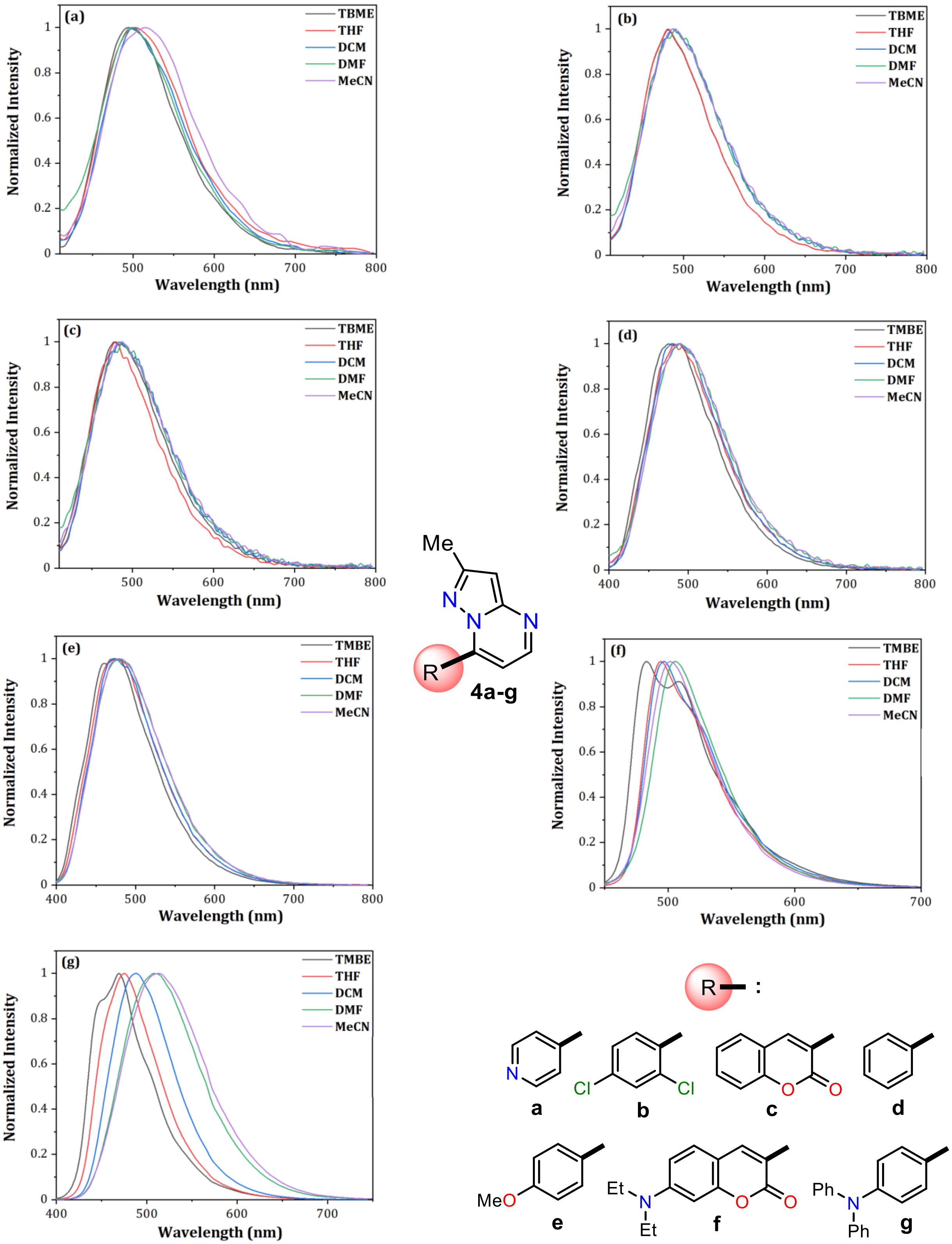

Fig. S2. Normalized emission spectra in different solvents (1 × 10^{-5} M at 20°C) and structures of compounds **4a-g**.

4.5 Emission spectra of compounds 4a-g in aqueous solutions

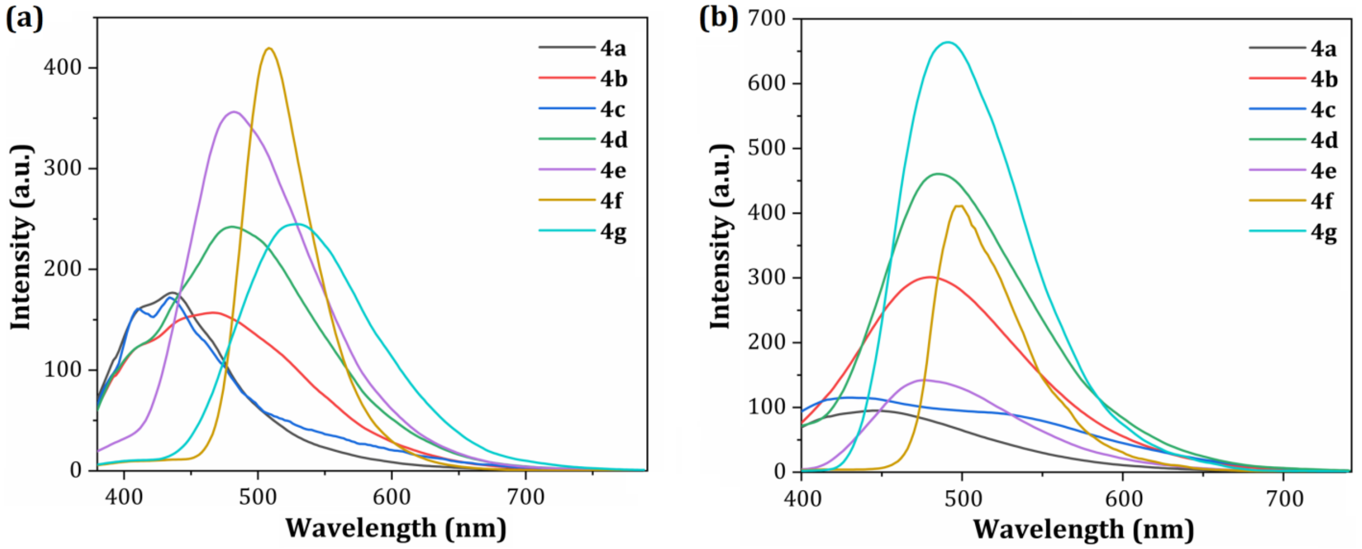

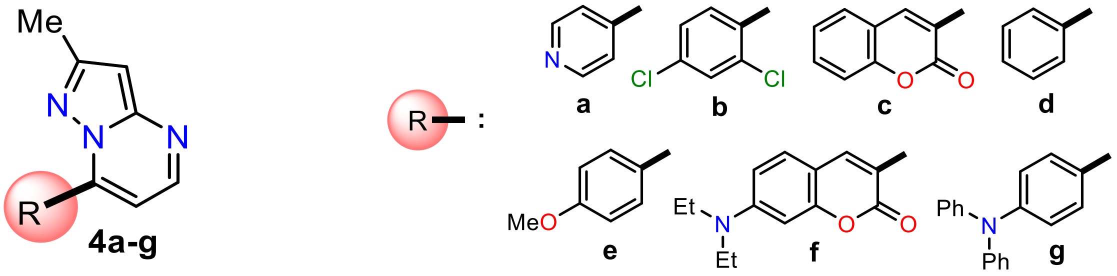

Fig. S3. Emission spectra of fluorophores **4a-g** in (a) ethanol-water 4:1 and (b) THF-water 4:1 (1 × 10^{-5} M at 20 °C).

5. Copies of NMR Spectra

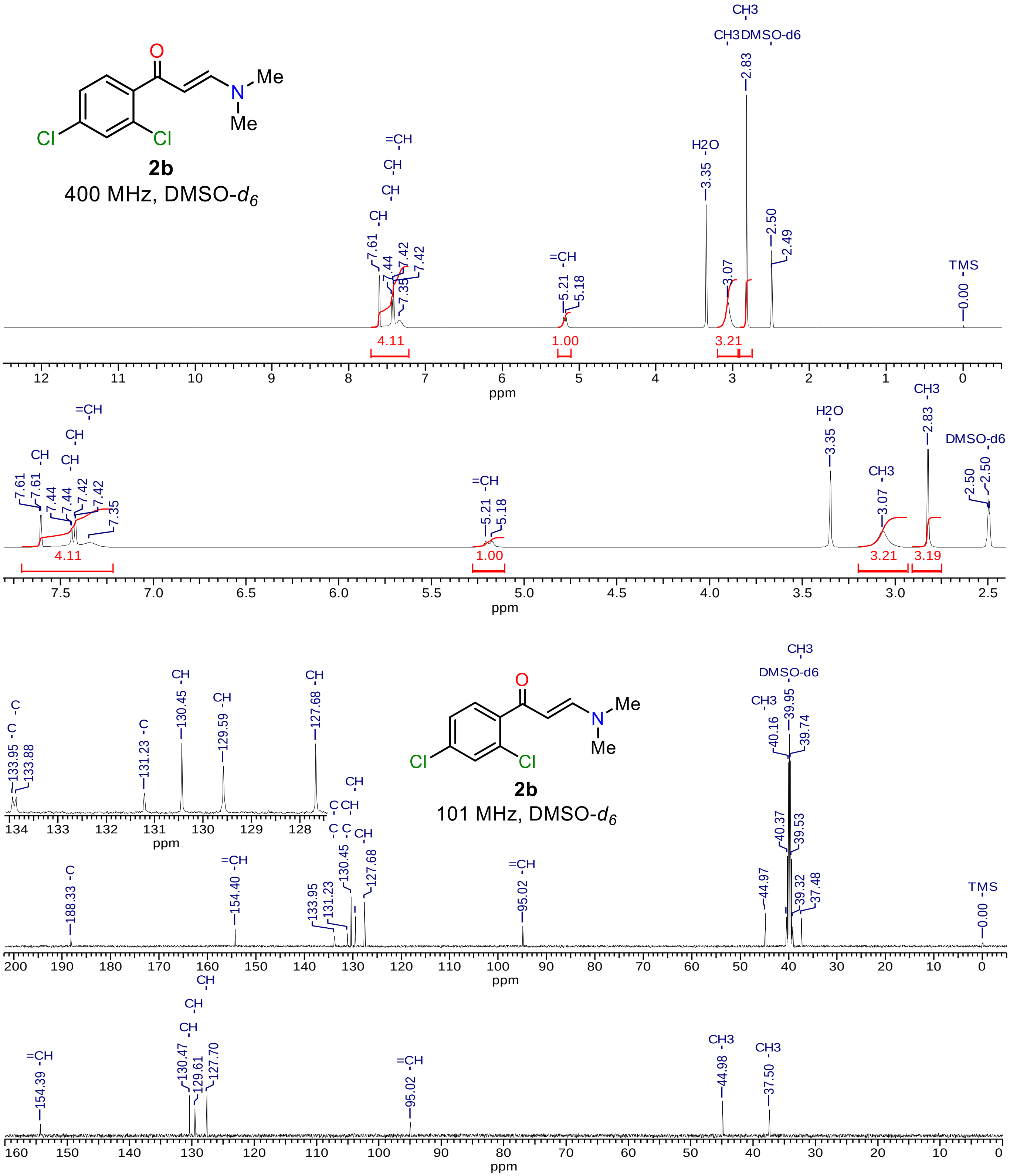

Fig. S4. ^{1}H and ^{13}C{^{1}H} NMR spectra of (*E*)-1-(2,4-dichlorophenyl)-3-(dimethylamino)prop-2-en-1-one (**2b**)

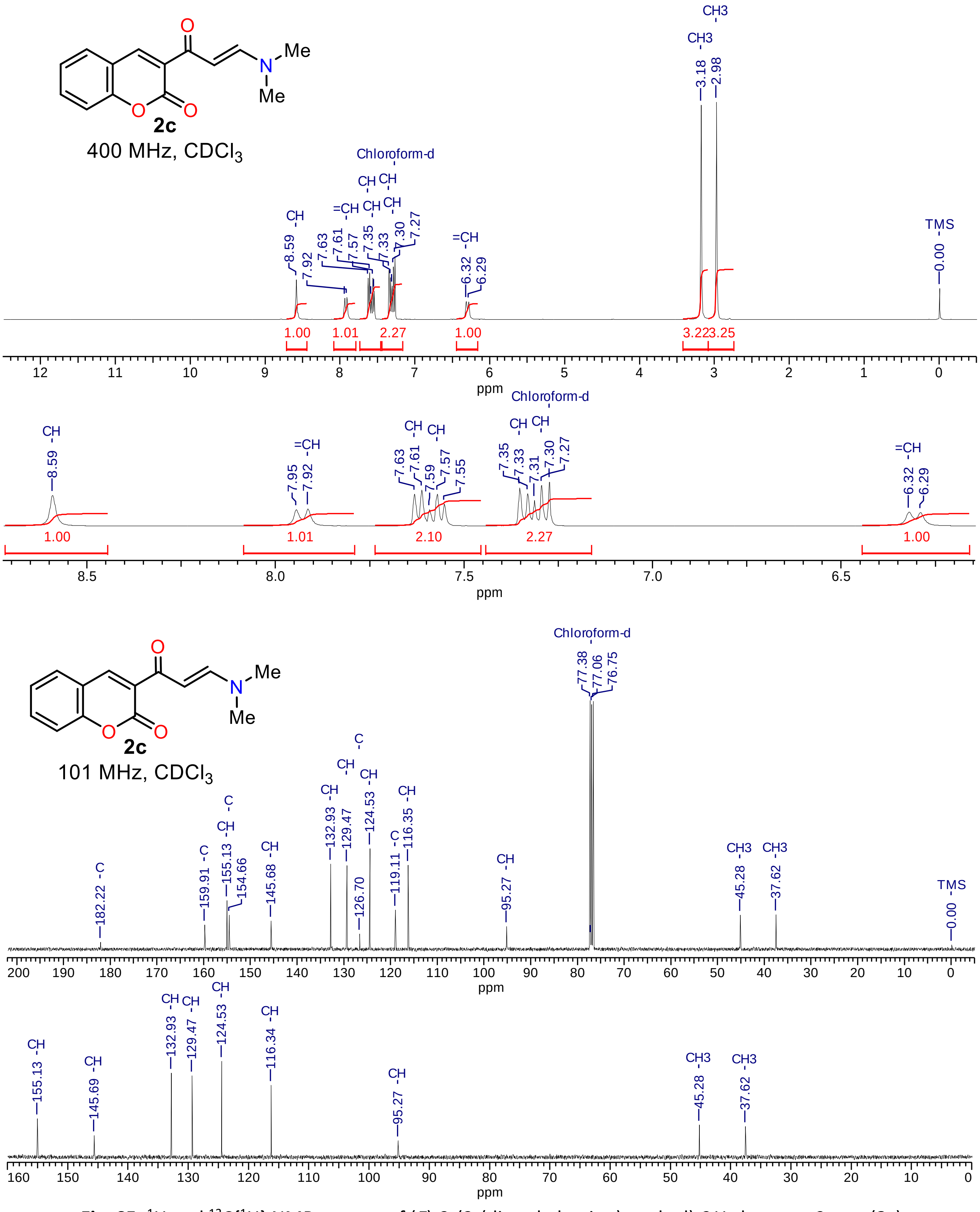

Fig. S5. ^{1}H and $^{13}C\{^{1}H\}$ NMR spectra of (*E*)-3-(3-(dimethylamino)acryloyl)-2*H*-chromen-2-one (**2c**)

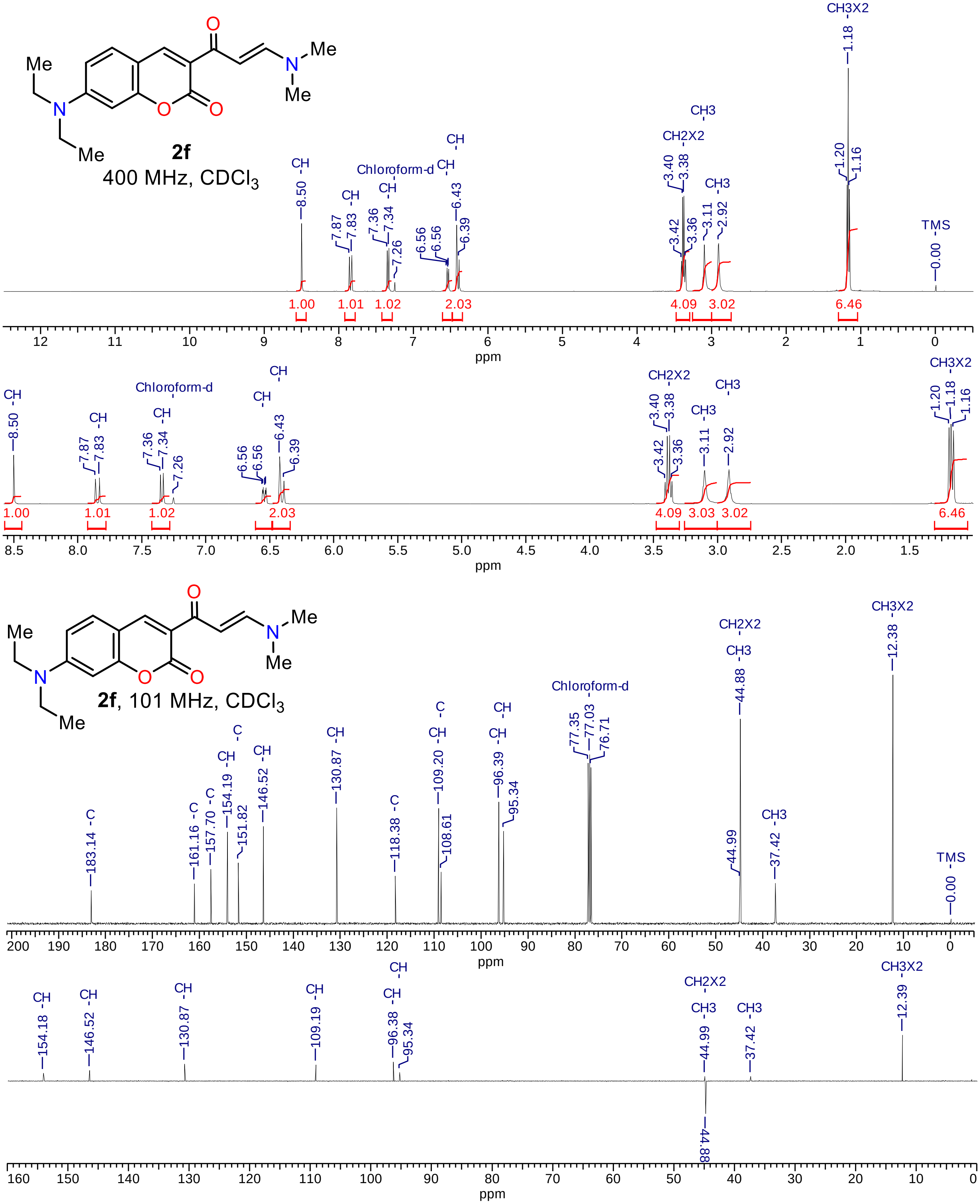

Fig. S6. ^{1}H and ^{13}C{^{1}H} NMR spectra of (*E*)-7-(diethylamino)-3-(3-(dimethylamino)acryloyl)-2*H*-chromen-2-one (**2f**)

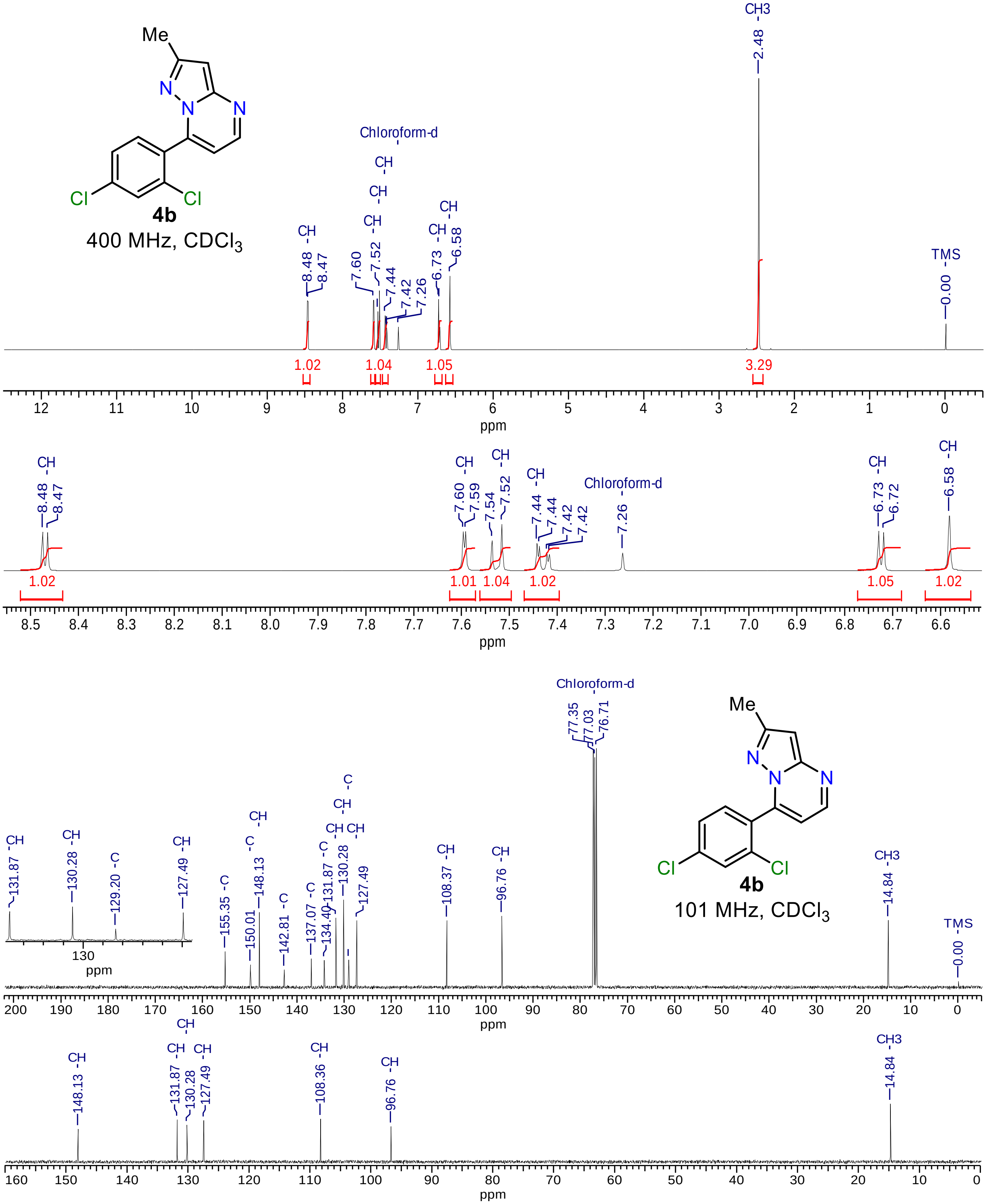

Fig. S7. ^{1}H and $^{13}C\{^{1}H\}$ NMR spectra of 7-(2,4-dichlorophenyl)-2-methylpyrazolo[1,5-*a*]pyrimidine (**4b**)

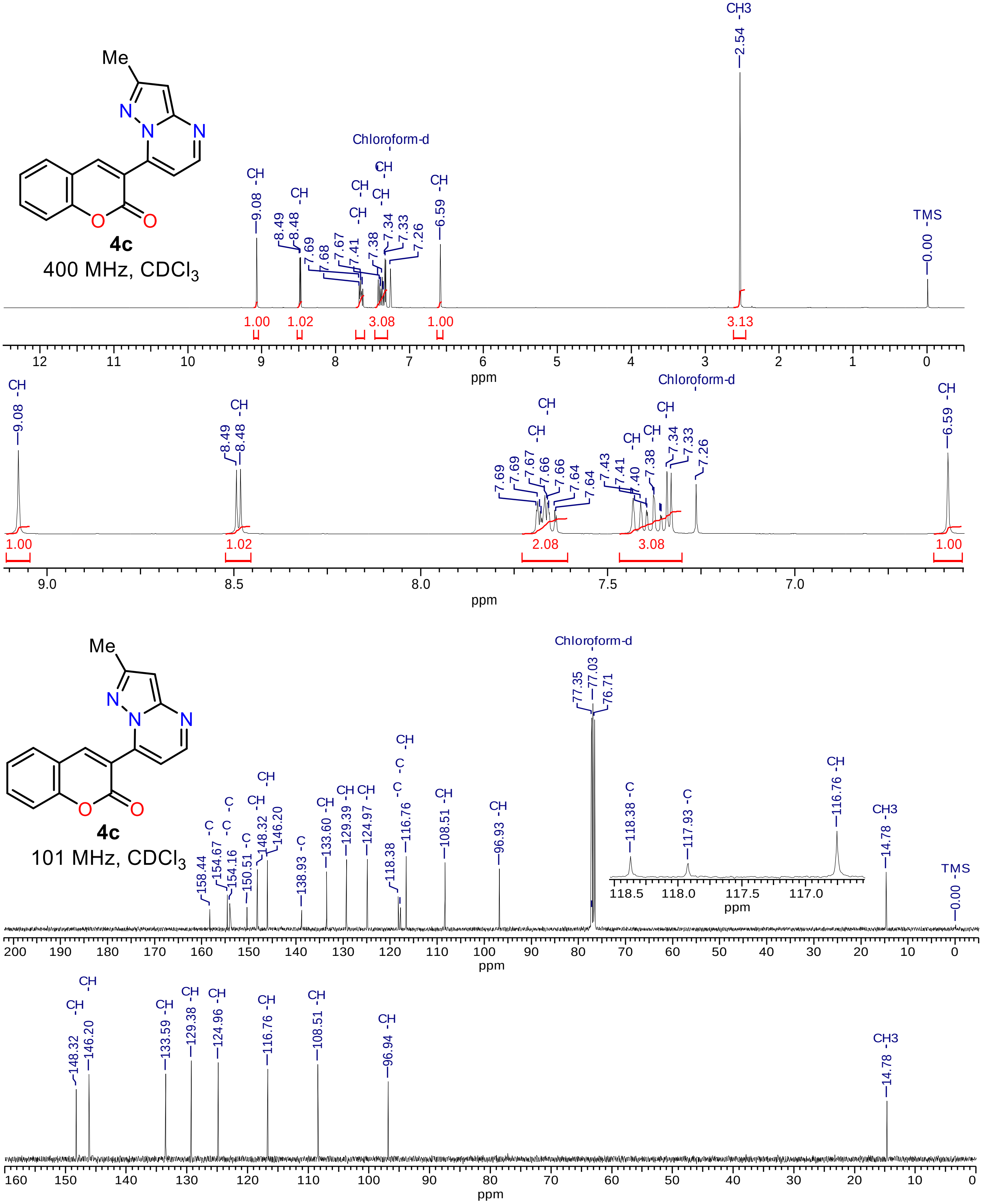

Fig. S8. ^{1}H and $^{13}C\{^{1}H\}$ NMR spectra of 3-(2-methylpyrazolo[1,5-*a*]pyrimidin-7-yl)-2*H*-chromen-2-one (**4c**)

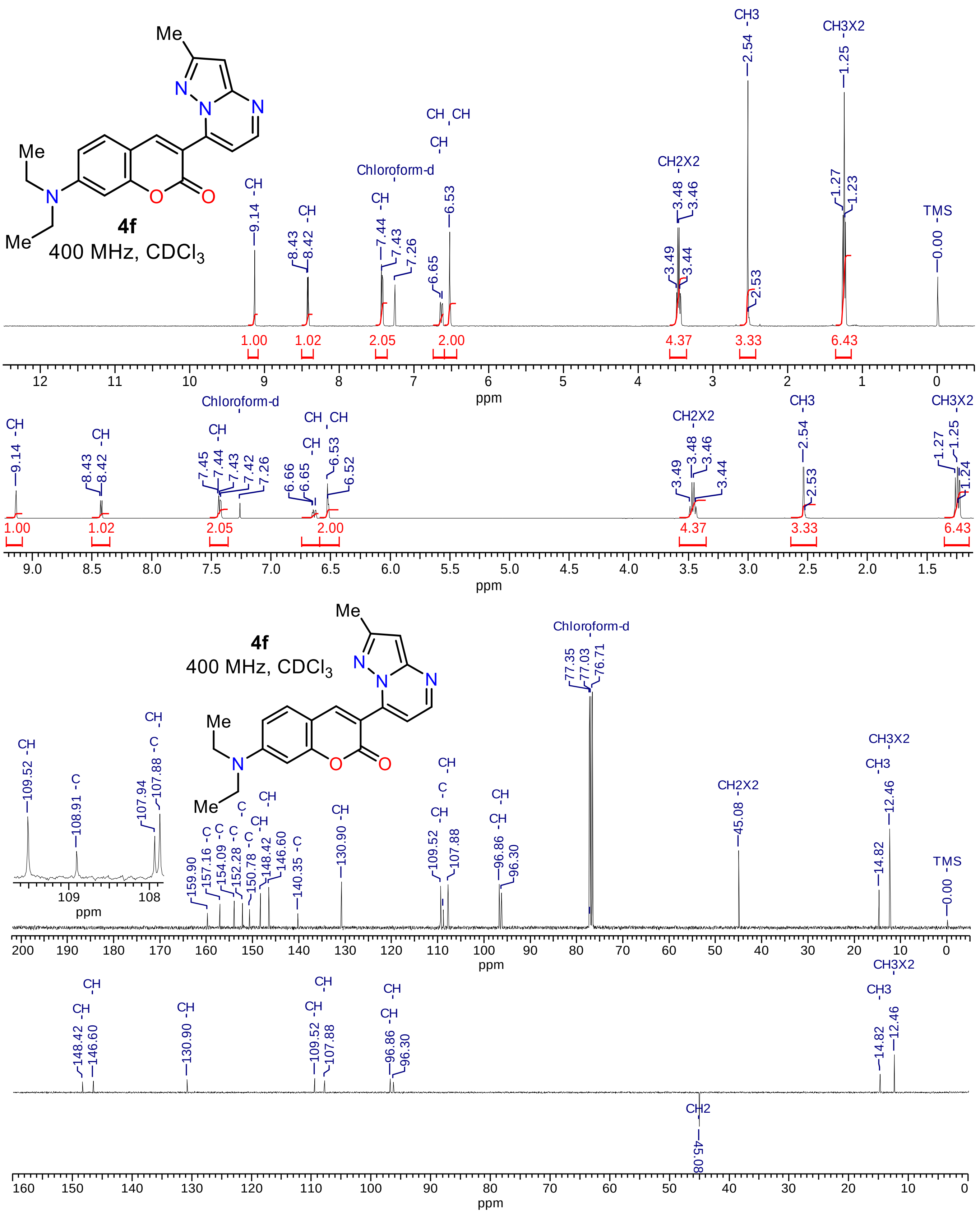

Fig. S9. ^{1}H and ^{13}C{^{1}H} NMR spectra of 7-(diethylamino)-3-(2-methylpyrazolo[1,5-*a*]pyrimidin-7-yl)-2*H*-chromen-2-one (**2f**)

6. HRMS analysis

Qualitative Analysis Report

**Data Filename**	24Cl enaminona 2.d	**Sample Name**	24Cl enaminona 2
**Sample Type**	Sample	**Position**	P1-F5
**Instrument Name**	Instrument 1	**User Name**	
**Acq Method**	Default 2019 Resolution.m	**Acquired Time**	11/27/2019 9:07:43 AM
**IRM Calibration Status**	Success	**DA Method**	Metodo-analisis-signaltonoise.m
**Comment**			
**Sample Group** **Info.**			

User Chromatograms

Fragmentor Voltage 175 **Collision Energy** 0 **Ionization Mode** ESI

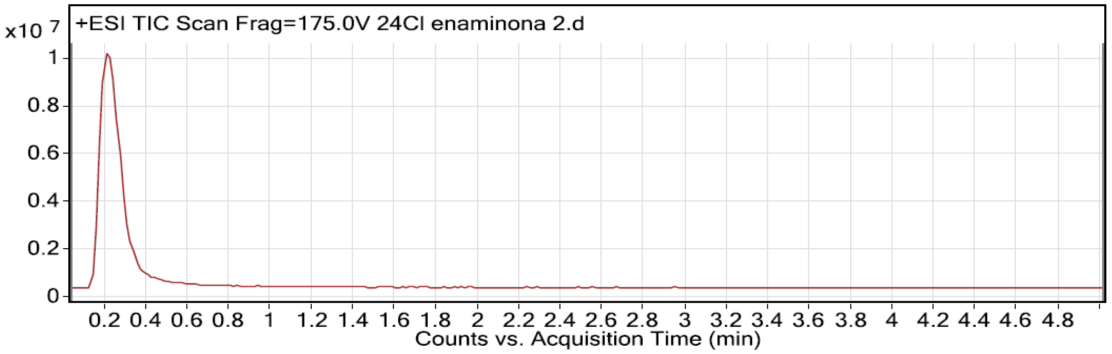

User Spectra

Fragmentor Voltage 175 **Collision Energy** 0 **Ionization Mode** ESI

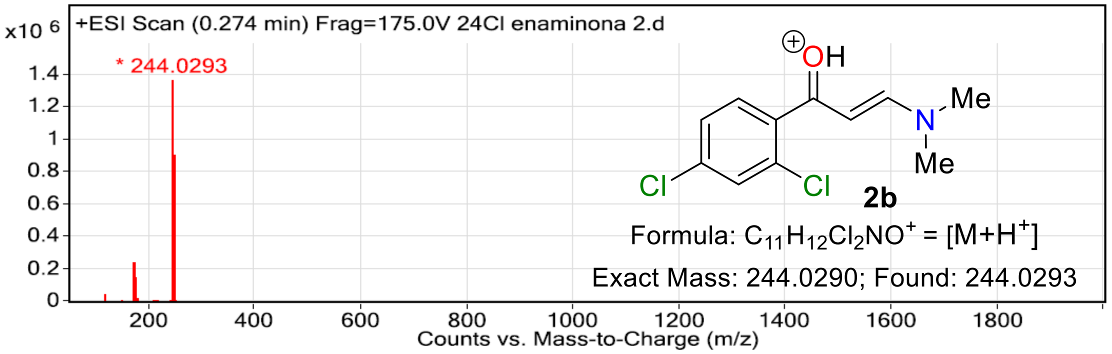

Peak List

m/z	z	Abund
172.9552		244604.1
174.9527		153042
244.0293	1	1367240.4
244.1073		72192.1
244.1526		74119
245.0304	1	157443.4
246.0259	1	904217.3
247.0287	1	104149.3
248.0222	1	129274.1

Fig. S10. HRMS analysis of *β*-enaminone **2b**

Qualitative Analysis Report

**Data Filename**	2.d	**Sample Name**	2
**Sample Type**	Sample	**Position**	P1-A2
**Instrument Name**	Instrument 1	**User Name**	
**Acq Method**	Default 2019 Resolution.m	**Acquired Time**	11/22/2019 10:30:54 AM
**IRM Calibration Status**	Success	**DA Method**	Metodo-analisis-signaltonoise.m
**Comment**			

Sample Group **Info.**

User Chromatograms

Fragmentor Voltage 175 **Collision Energy** 0 **Ionization Mode** ESI

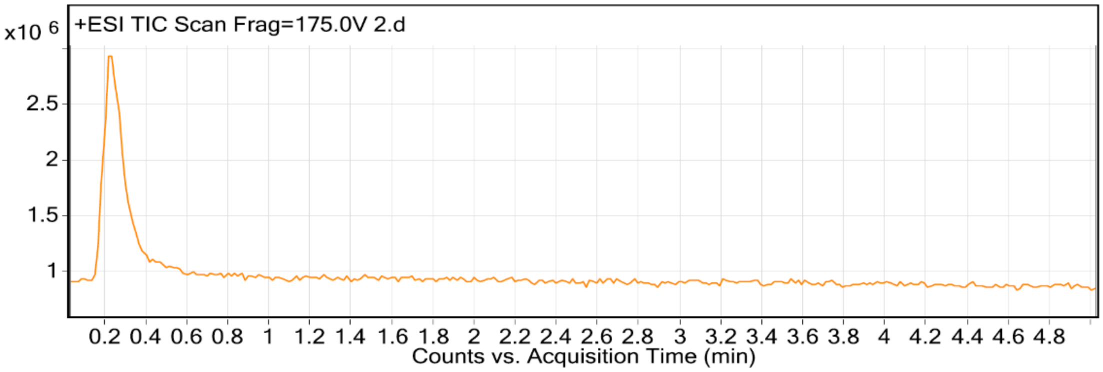

User Spectra

**Fragmentor Voltage**	**Collision Energy**	**Ionization Mode**
175	0	ESI

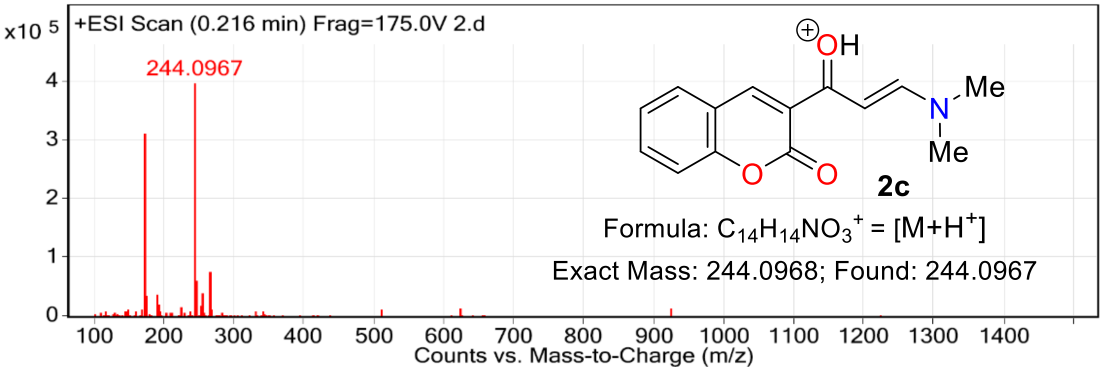

Peak List

m/z	z	Abund
171.0433		56253.5
173.0226	1	311849
173.0533		28255.4
174.0251	1	34708.6
189.0539		36921
244.0967	1	397189
244.1726		25512.5
245.0998	1	61580.6
255.2682		39656.3
266.0785		76540.5

Fig. S11. HRMS analysis of *β*-enaminone **2c**

Qualitative Analysis Report

**Data Filename**	N19.d	**Sample Name**	Sample19
**Sample Type**	Sample	**Position**	P1-C1
**Instrument Name**	Instrument 1	**User Name**	
**Acq Method**	Default 2019 Resolution.m	**Acquired Time**	9/23/2019 4:17:52 PM
**IRM Calibration Status**	Success	**DA Method**	Metodo-analisis-signaltonoise.m
**Comment**			

Sample Group **Info.**

User Chromatograms

Fragmentor Voltage 175 **Collision Energy** 0 **Ionization Mode** ESI

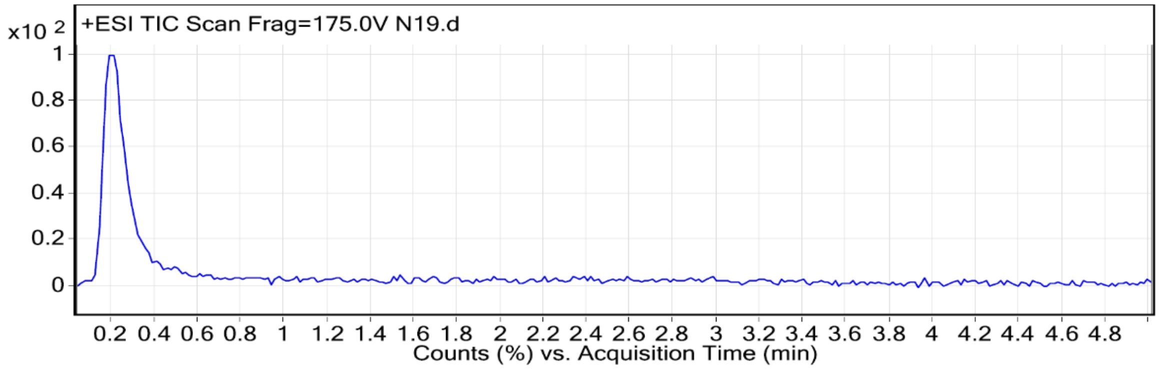

User Spectra

Fragmentor Voltage	Collision Energy	Ionization Mode
175	0	ESI

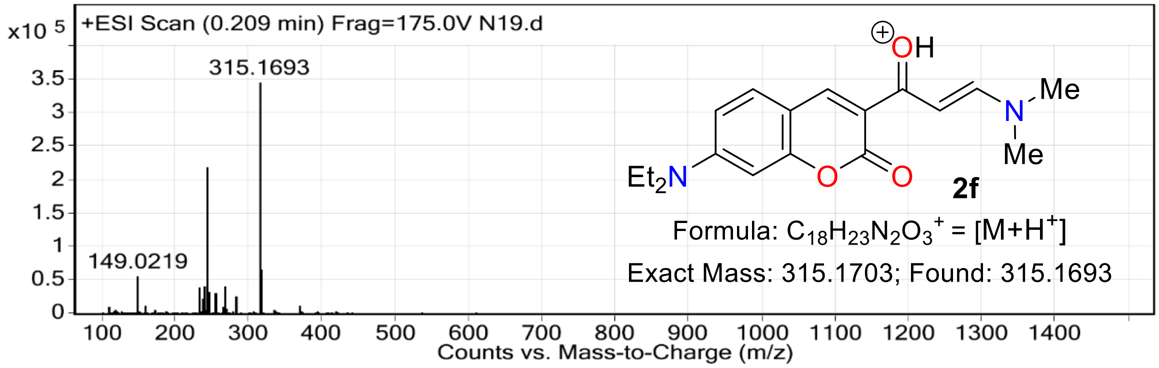

Peak List

m/z	z	Abund
149.0219		55966.6
232.1244		38771
240.1112		41872.3
244.0953	1	219268.5
245.0961	1	32628.4
254.1071		30076.8
268.1425		42245.3
282.1583		27474.8
315.1693	1	346514.3
316.1721	1	65398.2

Fig. S12. HRMS analysis of *β*-enaminone **2f**

Qualitative Analysis Report

**Data Filename**	PPirminCH324ClPh8.d	**Sample Name**	PPirminCH324ClPh
**Sample Type**	Sample	**Position**	P2-A8
**Instrument Name**	Instrument 1	**User Name**	
**Acq Method**	Default 2019 Resolution.m	**Acquired Time**	9/27/2019 7:34:19 PM
**IRM Calibration Status**	Success	**DA Method**	Metodo-analisis-signaltonoise.m
**Comment**			

Sample Group **Info.**

User Chromatograms

Fragmentor Voltage 175 **Collision Energy** 0 **Ionization Mode** ESI

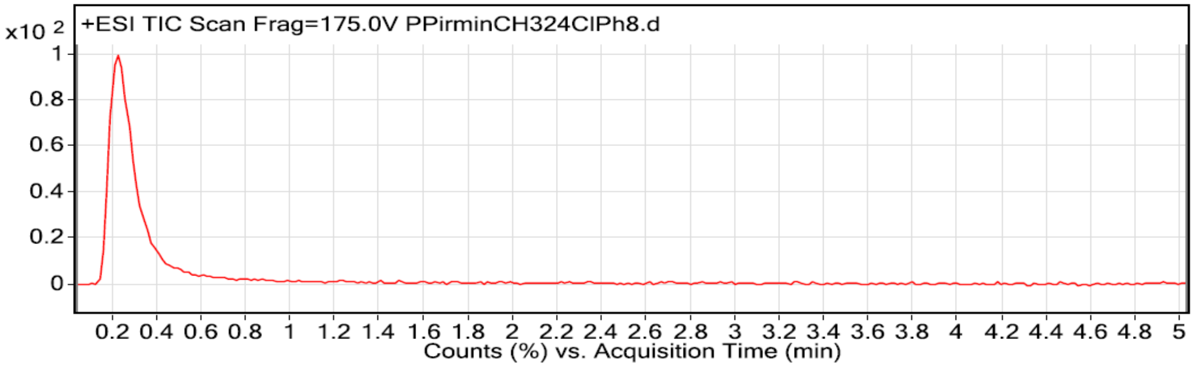

User Spectra

Fragmentor Voltage 175 **Collision Energy** 0 **Ionization Mode** ESI

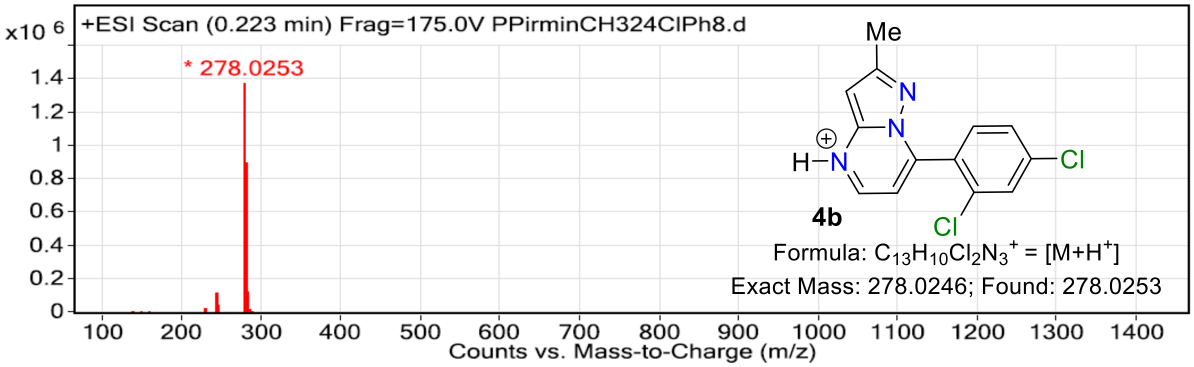

Peak List

m/z	z	Abund
242.0472		121510.5
278.0253	1	1379075.9
278.1074		71750.2
278.1553		71875.4
279.0266	1	190448.6
280.022	1	899165.3
281.0233	1	126049.7
282.0183	1	129409.5

Fig. S13. HRMS analysis of the 2-methylpyrazolo[1,5-*a*]pyrimidine **4b**

Qualitative Analysis Report

**Data Filename**	N13.d	**Sample Name**	Sample13
**Sample Type**	Sample	**Position**	P1-B4
**Instrument Name**	Instrument 1	**User Name**	
**Acq Method**	Default 2019 Resolution.m	**Acquired Time**	9/23/2019 3:45:05 PM
**IRM Calibration Status**	Success	**DA Method**	Metodo-analisis-signaltonoise.m
**Comment**			

Sample Group **Info.**

User Chromatograms

Fragmentor Voltage 175 **Collision Energy** 0 **Ionization Mode** ESI

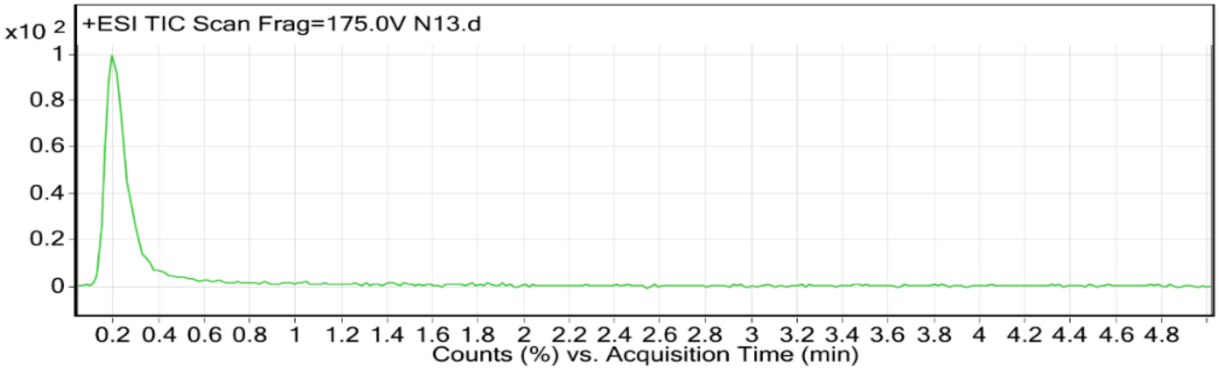

User Spectra

Fragmentor Voltage	Collision Energy	Ionization Mode
175	0	ESI

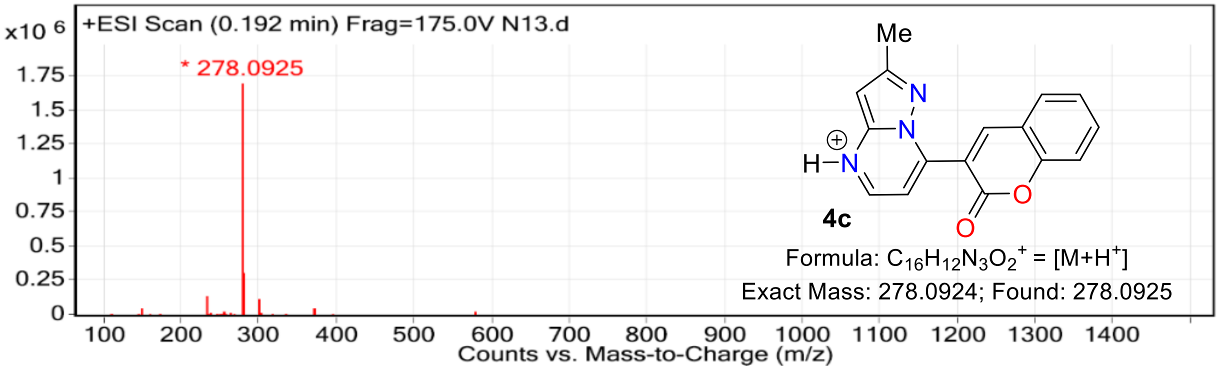

Peak List

m/z	z	Abund
232.1247		145559.8
278.0925	1	1705738.4
278.2048		90085.6
279.0942	1	319566.7
300.0732		123029

Fig. S14. HRMS analysis of the 2-methylpyrazolo[1,5-*a*]pyrimidine **4c**

Qualitative Analysis Report

**Data Filename**	N20.d	**Sample Name**	Sample20
**Sample Type**	Sample	**Position**	P1-C2
**Instrument Name**	Instrument 1	**User Name**	
**Acq Method**	Default 2019 Resolution.m	**Acquired Time**	9/23/2019 4:23:18 PM
**IRM Calibration Status**	Success	**DA Method**	Metodo-analisis-signaltonoise.m
**Comment**			

Sample Group **Info.**

User Chromatograms

Fragmentor Voltage 175 **Collision Energy** 0 **Ionization Mode** ESI

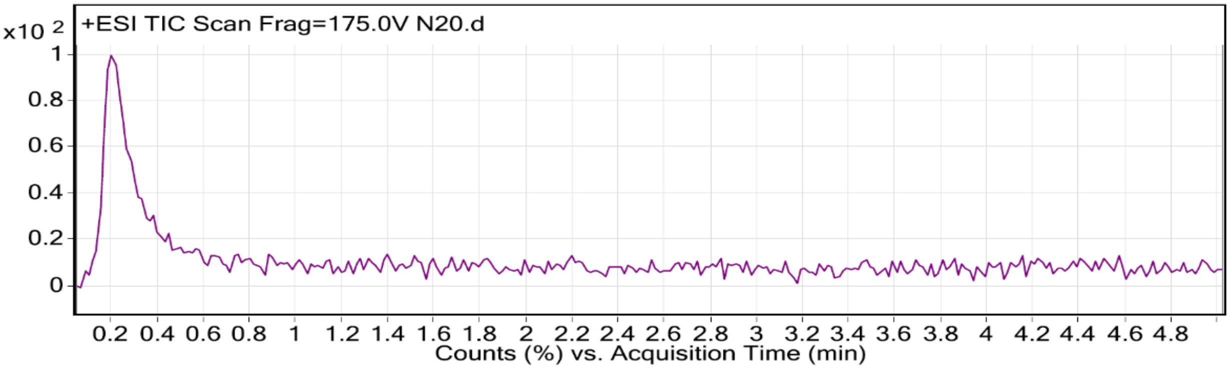

User Spectra

**Fragmentor Voltage**	**Collision Energy**	**Ionization Mode**
175	0	ESI

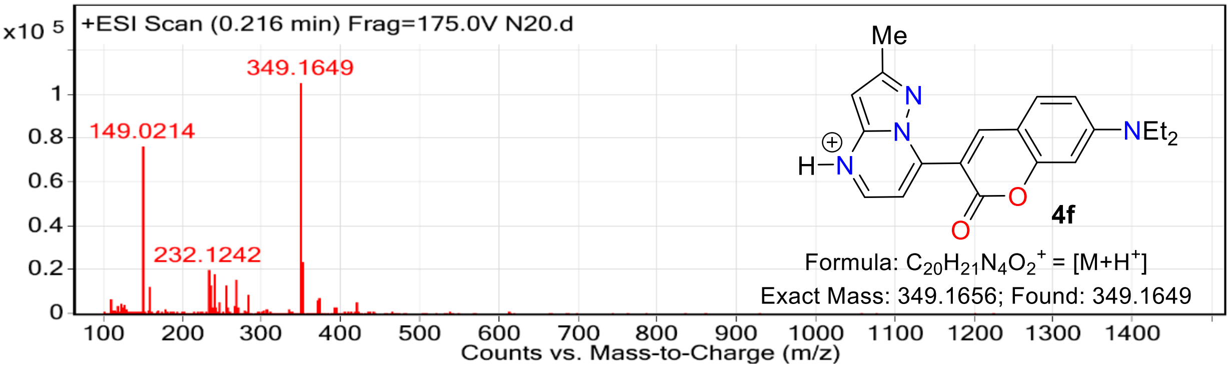

Peak List

m/z	z	Abund
149.0214	1	76771.2
158.1532		12777
232.1242		20247.5
236.115		13502.2
240.1107		18604.3
254.1057		13130
268.1421		15958.7
282.1558		9063.2
349.1649	1	105690.2
350.1666	1	24304.3

Fig. S15. HRMS analysis of the 2-methylpyrazolo[1,5-*a*]pyrimidine **4f**

7. Green metrics calculations

For cost per gram calculations the Sigma-Aldrich prices of on-line catalog was used. The reaction mass efficiency was calculated using the equation S3.

$$RME = \frac{(mass\ of\ product\ C)}{(mass\ of\ A\ + mass\ of\ B \ldots)} \qquad \text{Equation S3.}$$

Table S1. Reaction mass efficiency and raw materials cost per gram calculations for **4a**.

Raw material	Reaction 1 (mass used, mg)	Reaction 2 (mass used, mg)	Total (mass used, mg)	Cost (USD)
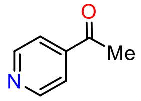 Chemical Formula: C_7H_7NO Exact Mass: 121.0528	121	-	121	0.13
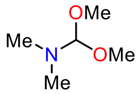 Chemical Formula: $C_5H_{13}NO_2$ Exact Mass: 119.0946	179	-	179	0.14
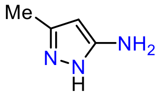 Chemical Formula: $C_4H_7N_3$ Exact Mass: 97.0640	-	96	96	0.22
**4a Mass obtained:** 185 mg	**RME**	46.7%	Total cost/g (USD)	2.7

Table S2. Reaction mass efficiency and raw materials cost per gram calculations for **4b**.

Raw material	Reaction 1 (mass used, mg)	Reaction 2 (mass used, mg)	Total (mass used, mg)	Cost (USD)
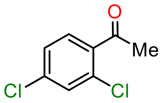 Chemical Formula: $C_8H_6Cl_2O$ Exact Mass: 187.9796	187	-	187	0.02
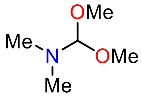 Chemical Formula: $C_5H_{13}NO_2$ Exact Mass: 119.0946	179	-	179	0.14
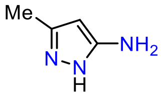 Chemical Formula: $C_4H_7N_3$ Exact Mass: 97.0640	-	82	82	0.19
**4b Mass obtained:** 185 mg	**RME**	40.0%	Total cost/g (USD)	1.9

Table S3. Reaction mass efficiency and raw materials cost per gram calculations for **4c**.

Raw material	Reaction 1 (mass used, mg)	Reaction 2 (mass used, mg)	Total (mass used, mg)	Cost (USD)
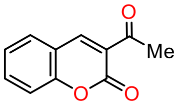 Chemical Formula: $C_{11}H_8O_3$ Exact Mass: 188.0473	188	-	188	0.85
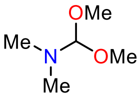 Chemical Formula: $C_5H_{13}NO_2$ Exact Mass: 119.0946	179	-	179	0.14
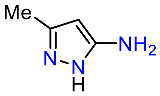 Chemical Formula: $C_4H_7N_3$ Exact Mass: 97.0640	-	84	84	0.19
**4c Mass obtained:** 194 mg	**RME**	43.0 %	Total cost/g (USD)	6.1

Table S4. Reaction mass efficiency and raw materials cost per gram calculations for **4d**.

Raw material	Reaction 1 (mass used, mg)	Reaction 2 (mass used, mg)	Total (mass used, mg)	Cost (USD)
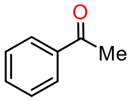 Chemical Formula: C_8H_8O Exact Mass: 120.0575	120	-	120	0.005
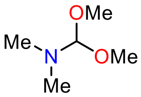 Chemical Formula: $C_5H_{13}NO_2$ Exact Mass: 119.0946	179	-	179	0.14
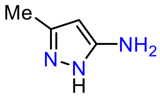 Chemical Formula: $C_4H_7N_3$ Exact Mass: 97.0640	-	94	94	0.22
**4d Mass obtained:** 195 mg	**RME**	49.6 %	Total cost/g (USD)	1.9

Table S5. Reaction mass efficiency and raw materials cost per gram calculations for **4e**.

Raw material	Reaction 1 (mass used, mg)	Reaction 2 (mass used, mg)	Total (mass used, mg)	Cost (USD)
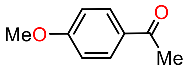 Chemical Formula: $C_9H_{10}O_2$ Exact Mass: 150.0681	150	-	150	0.02
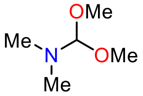 Chemical Formula: $C_5H_{13}NO_2$ Exact Mass: 119.0946	179	-	179	0.14
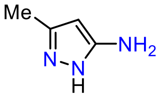 Chemical Formula: $C_4H_7N_3$ Exact Mass: 97.0640	-	92	92	0.21
**4e Mass obtained:** 216 mg	**RME**	51.3 %	Total cost/g (USD)	1.7

Table S6. Reaction mass efficiency and raw materials cost per gram calculations for **4f**.

Raw material	Reaction 1 (mass used, mg)	Reaction 2 (mass used, mg)	Total (mass used, mg)	Cost (USD)
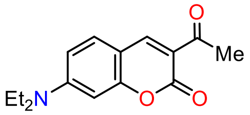 Chemical Formula: $C_{15}H_{17}NO_3$ Exact Mass: 259.1208	259	-	259	23.75
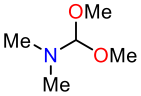 Chemical Formula: $C_5H_{13}NO_2$ Exact Mass: 119.0946	179	-	179	0.14
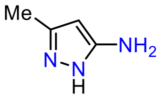 Chemical Formula: $C_4H_7N_3$ Exact Mass: 97.0640	-	81	81	0.19
**4f Mass obtained:** 251 mg	**RME**	48.4 %	Total cost/g (USD)	95.9

Table S7. Reaction mass efficiency and raw materials cost per gram calculations for **4g**.

Raw material	Reaction 1 (mass used, mg)	Reaction 2 (mass used, mg)	Total (mass used, mg)	Cost (USD)
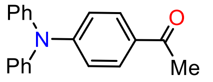 Chemical Formula: $C_{20}H_{17}NO$ Exact Mass: 287.1310	287	-	287	18.80
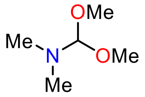 Chemical Formula: $C_5H_{13}NO_2$ Exact Mass: 119.0946	179	-	179	0.14
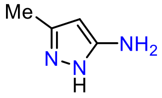 Chemical Formula: $C_4H_7N_3$ Exact Mass: 97.0640	-	83	83	0.19
**4g Mass obtained:** 291 mg	**RME**	53.0 %	Total cost/g (USD)	65.7

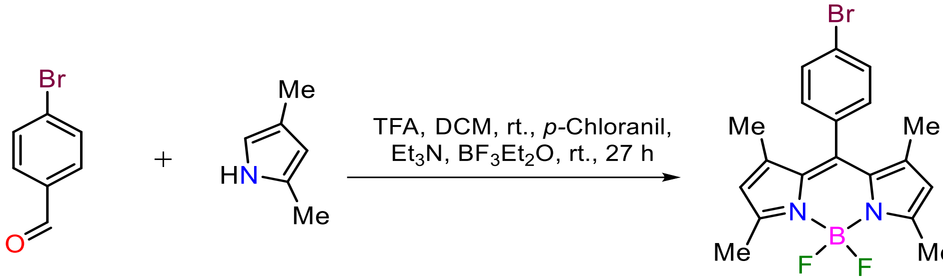

BODIPY-1 (19 %)

Scheme S3. Synthesis of **BODIPY-1**.

Table S8. Reaction mass efficiency and raw materials cost per gram calculations for BODIPY-1[9].

Raw material	Reaction 1 (mass, mg)		Cost (USD)	
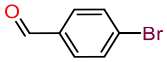 Chemical Formula: C_7H_5BrO Exact Mass: 183.9524	184		0.55	
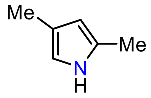 Chemical Formula: C_6H_9N Exact Mass: 95.0735	210		6.76	
Trifluoroacetic acid	75		0.009	
Tetrachloro-1,4-benzoquinone	123		0.12	
$BF_3{\cdot}OEt_2$	3358		0.015	
Temperature	rt. (27 h)		-	
**BODIPY-1, Mass obtained**: 76 mg	**RME**	1.31%	Total cost/g (USD)	98.0

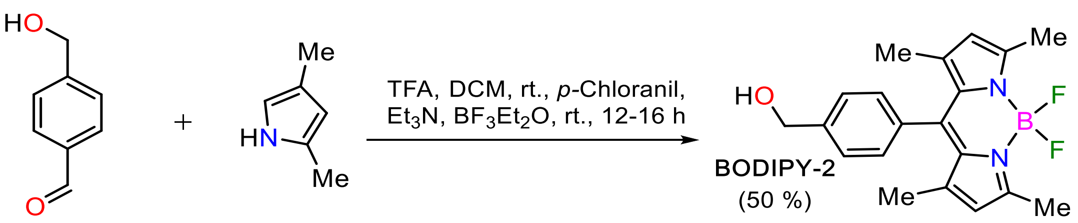

Scheme S4. Synthesis of **BODIPY-2**.

Table S9. Reaction mass efficiency and raw materials cost per gram calculations for **BODIPY-2**[10].

**Raw material**	**Reaction 1 (mass used, mg)**		**Cost (USD)**	
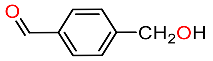 Chemical Formula: $C_8H_8O_2$ Exact Mass: 136.0524	140		5.57	
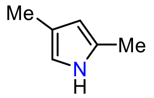 Chemical Formula: C6H9N Exact Mass: 95.0735	7.5		0.004	
Trifluoroacetic acid	7.5		0.004	
Tetrachloro-1,4-benzoquinone	230		0.35	
BF_3·OEt_2	3450		0.41	
Temperature	rt. (12-16 h)			
**BODIPY-2, Mass obtained:** 177 mg	**RME**	4.37%	Total cost/g	35.8

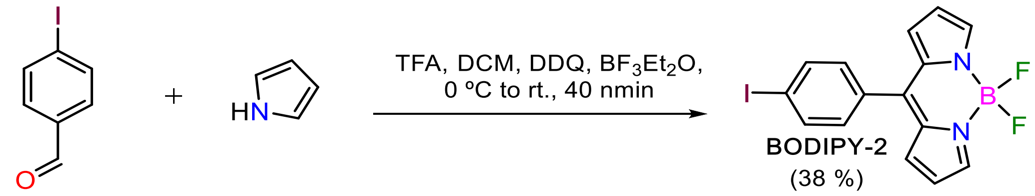

Scheme S5. Synthesis of **BODIPY-3**.

Table S10. Reaction mass efficiency and raw materials cost per gram calculations for **BODIPY-3**[11].

**Raw material**	**Reaction 1 (mass used, mg)**		**Cost (USD)**	
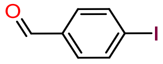 Chemical Formula: C_7H_5IO Exact Mass: 231.9385	231		24.02	
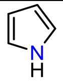 Chemical Formula: C_4H_5N Exact Mass: 67.0422	135		0.11	
Trifluoroacetic acid	35		0.004	
2,3-dichloro-5,6-dicyanobenzoquinone (DDQ)	60		0.21	
BF_3·OEt_2	376		0.05	
Temperature	0 °C (40 min)			
**BODIPY-3, Mass obtained:** 150 mg	**RME**	17.9%	Total cost/g (USD)	162.6

8. Computational details

The geometries of compounds **4a-g** were optimized at the B3LYP level of theory[10] with the Ahlrichs def2-TZVP basis set (in the resolution of identity approach has been used the def2/J and def2-TZVP/C auxiliary basis sets for Coulomb and correlation integral calculations, respectively),[11-13] as implemented in the ORCA 4.2.0 package.[14,15] It has also been included the long range dispersion correction (as developed by Grimme and included in ORCA by the D3BJ approximation)[16] and the implicit solvent effects by the Conductor-like Polarizable Continuum Model (CPCM).[17] The respective dielectric constants and refractive index of each solvent are as follow: MTBE (2.60 and 1.369), THF (7.25 and 1.407), DCM (9.08 and 1.424), DMF (38.30 and 1.430), and ACN (36.60 and 1.344). The threshold for the energy convergence in the self-consistent field procedure was 1x10^{-8} a.u. No negative normal modes were obtained by analytical frequency calculations on the optimized geometries. Additionally, in order to predict the absorption and emission spectra, TD-DFT calculations were performed on the lowest five singlet excited states, combined with a path integral approach to the dynamics of the transitions (the ESD module in ORCA) to incorporate the vibronic couplings.[18,19] The simplest Vertical Gradient (VG) approximation was used to estimate the excited state geometries and Hessians, and a Gaussian fitting with a line width of 50 nm was used for plotting the spectra curves. Avogadro visualization tool for plotting geometries and orbitals was used.[20]

Table S11. Properties of compound **4a** in the singlet ground state as function of the solvents.

Property	DMF	CCN	DCM	THF	MTB	VAC
Charge N4	-0.273	-0.273	-0.265	-0.263	-0.243	-0.201
Charge C3	-0.273	-0.273	-0.267	-0.266	-0.250	-0.215
C7-C8 bond	1.473	1.473	1.473	1.473	1.473	1.473
Dihedral	-40.000	-40.000	-39.600	-39.500	-38.300	-35.600
Polarizability	169.602	169.604	169.729	169.765	170.109	170.845
H-L Gap	4.075	4.074	4.067	4.065	4.046	4.014

Table S12. Properties of compound **4b** in the singlet ground state as function of the solvents.

Property	DMF	CCN	DCM	THF	MTB	VAC
Charge N4	-0.275	-0.275	-0.267	-0.265	-0.245	-0.203
Charge C3	-0.280	-0.280	-0.276	-0.275	-0.261	-0.230
C7-C8 bond	1.476	1.477	1.476	1.476	1.476	1.475
Dihedral	-67.200	-67.200	-64.700	-63.900	-60.800	-56.100
Polarizability	200.826	200.825	201.378	201.557	202.359	203.721
H-L Gap	4.392	4.392	4.347	4.332	4.268	4.170

Table S13. Properties of compound **4c** in the singlet ground state as function of the solvents.

Property	DMF	CCN	DCM	THF	MTB	VAC
Charge N4	-0.274	-0.274	-0.266	-0.264	-0.243	-0.200
Charge C3	-0.278	-0.278	-0.273	-0.272	-0.257	-0.221
C7-C8 bond	1.468	1.468	1.468	1.468	1.468	1.469
Dihedral	-39.400	-39.400	-37.900	-37.400	-34.000	-26.700
Polarizability	229.769	229.774	230.273	230.416	231.630	234.220
H-L Gap	3.614	3.614	3.591	3.584	3.533	3.456

Table S14. Properties of compound **4d** in the singlet ground state as function of the solvents.

Property	DMF	CCN	DCM	THF	MTB	VAC
Charge N4	-0.268	-0.280	-0.272	-0.270	-0.249	-0.206
Charge C3	-0.266	-0.279	-0.273	-0.271	-0.255	-0.219
C7-C8 bond	1.471	1.471	1.471	1.471	1.471	1.472
Dihedral	-40.600	-40.000	-40.000	-39.900	-38.700	-36.600
Polarizability	176.726	176.373	176.392	176.428	176.804	177.485
H-L Gap	4.212	4.229	4.225	4.222	4.189	4.139

Table S15. Properties of compound **4e** in the singlet ground state as function of the solvents.

Property	DMF	CCN	DCM	THF	MTB	VAC
Charge N4	-0.285	-0.284	-0.276	-0.274	-0.252	-0.208
Charge C3	-0.284	-0.284	-0.278	-0.276	-0.260	-0.223
C7-C8 bond	1.466	1.466	1.466	1.466	1.467	1.467
Dihedral	-36.000	-36.000	-35.900	-35.900	-35.300	-33.900
Polarizability	202.267	202.269	202.275	202.277	202.432	202.774
H-L Gap	4.146	4.145	4.145	4.145	4.136	4.117

Table S16. Properties of compound **4f** in the singlet ground state as function of the solvents.

Property	DMF	CCN	DCM	THF	MTB	VAC
Charge N4	-0.284	-0.283	-0.275	-0.272	-0.250	-0.205
Charge C3	-0.303	-0.303	-0.298	-0.296	-0.282	-0.246
C7-C8 bond	1.462	1.462	1.463	1.463	1.464	1.465
Dihedral	-36.100	-36.100	-35.000	-34.600	-31.800	-25.500
Polarizability	328.857	328.848	328.245	328.059	326.881	325.690
H-L Gap	3.276	3.276	3.293	3.299	3.333	3.364

Table S17. Properties of compound **4g** in the singlet ground state as function of the solvents.

Property	DMF	CCN	DCM	THF	MTB	VAC
Charge N4	-0.286	-0.286	-0.277	-0.275	-0.253	-0.208
Charge C3	-0.287	-0.287	-0.281	-0.279	-0.263	-0.226
C7-C8 bond	1.464	1.464	1.465	1.465	1.465	1.466
Dihedral	-34.300	-34.400	-34.200	-34.200	-33.500	-32.200
Polarizability	360.384	360.329	360.163	360.090	359.752	359.378
H-L Gap	3.459	3.460	3.476	3.481	3.516	3.568

We have also predicted the absorption and fluorescence spectra, including the all vibronic transitions, based on the excited singlet of interest of each compound. It was used the ground state Hessian and the excited state gradient to extrapolate the excited state geometry, then by using the same Hessian in the excited state, the energy differences were calculated. The obtained spectra are very close to the

experimental ones at 20 °C (Fig. S16). The transitions associated to the absorption spectra are consistent with the description above; neverthenless multiple peaks arises from vibronic couplings. Similarly, the fluorescence rates of all probes are caused by a relaxation process from the first excited singlet to the ground state, in addition they are dominated by a LUMO → HOMO electronic transition. As depicted in Fig. 16b, all calculated emissions are in the visible region (450–550 nm for **4a–b/d–g** and 550–650 for **4c**). Probes **4e**, **4f**, and **4g** showed the highest intensities (ca. 5.0 $\times 10^8$, 3.0 $\times 10^9$, and 1.5 $\times 10^9$, respectively) in which the photon emission is a result of a charge transfer from the PP fragment to 7-aryl groups (EDGs). Contrarily, probes **4a–d** showed lower emmision intensities (below to 2.5 × 10^8) by an inverse ICT process going from the 7-aryl groups (EWGs and NG) to the fused *N*-heterocyclic fragment. This estimated behavior agrees with the experimental data.

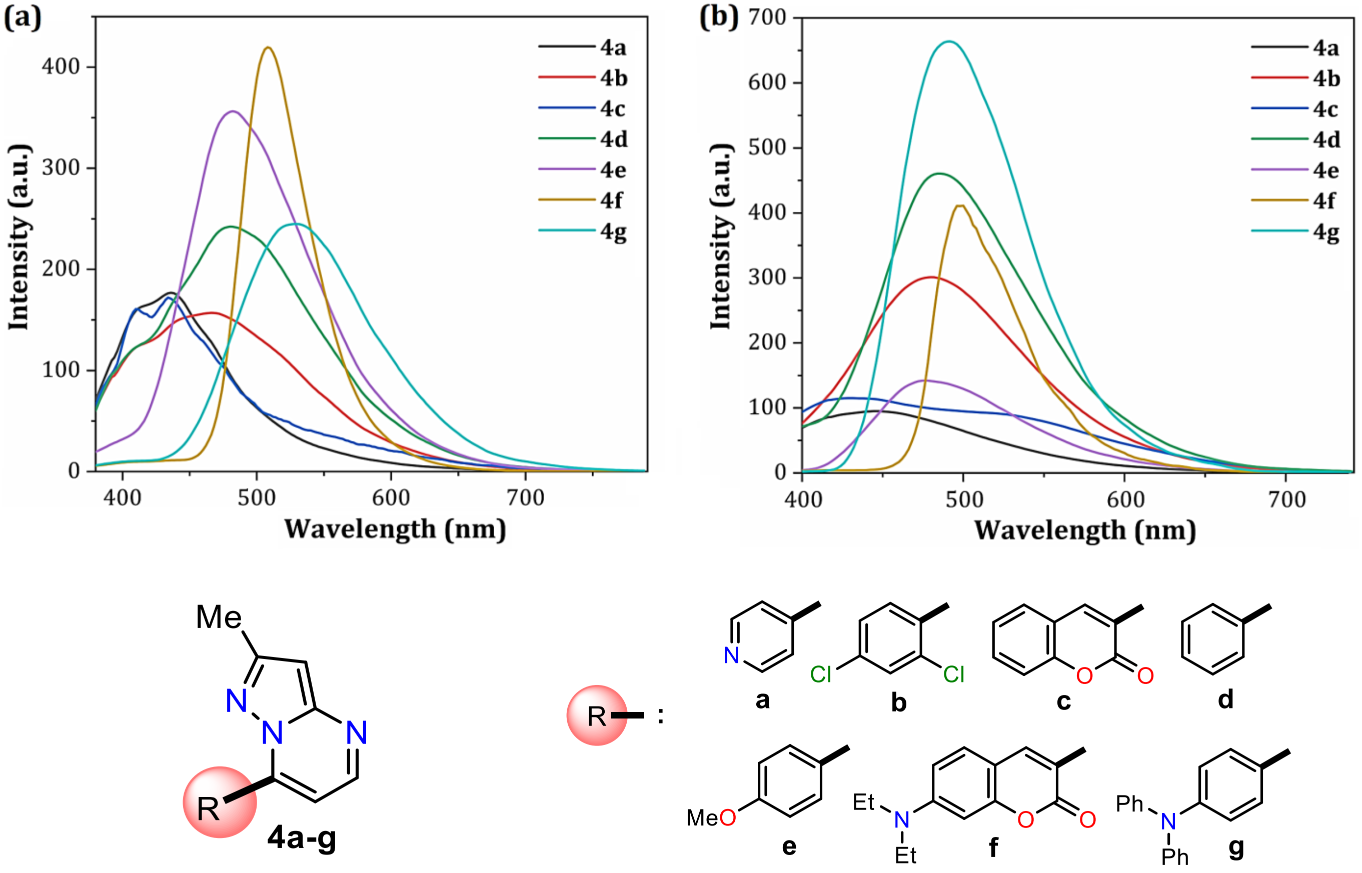

Fig. S16. Calculated absorption (a) and fluorescence (b) spectra of **4a–g** based on TD-DFT.

9. References

1. J.-C. Castillo, H.-A. Rosero and J. Portilla, *RSC Adv.*, 2017, **7**, 28483–28488.
2. F. M. A. A. El-Taweel and M. H. Elnagdi, *J. Heterocyclic Chem.*, 2001, **38**, 981–984.
3. S. K. Prajapti, A. Nagarsenkar, S. D. Guggilapu, K. K. Gupta, L. Allakonda, M. K. Jeengar, V. G. M. Naidu and B. N. Babu, *Bioorg. Med. Chem. Lett.*, 2016, **26**, 3024–3028.
4. A. Tigreros, M. Macías and J. Portilla, *Dye. Pigment.*, 2020, 108730.
5. C. Würth, M. Grabolle, J. Pauli, M. Spieles and U. Resch-Genger, *Nat. Protoc.*, 2013, **8**, 1535.
6. A. Tigreros, V. Dhas, A. Ortiz, B. Insuasty, N. Martín and L. Echegoyen, *Sol. Energy Mater. Sol. Cells*, 2014, **121**, 61–68.
7. L. Wang, G. Fang and D. Cao, *Sensors Actuators B Chem.*, 2015, **207**, 849–857.
8. C.-H. Lee, H.-J. Yoon, J.-S. Shim and W.-D. Jang, *Chem. – A Eur. J.*, 2012, **18**, 4513–4516.
9. R. Sukato, N. Sangpetch, T. Palaga, S. Jantra, V. Vchirawongkwin, C. Jongwohan, M. Sukwattanasinitt and S. Wacharasindhu, *J. Hazard. Mater.*, 2016, **314**, 277–285.
10. P. J. Stephens, F. J. Devlin, C. F. Chabalowski and M. J. Frisch, *J. Phys. Chem.*, 1994, **98**, 11623–11627.
11. F. Weigend and R. Ahlrichs, *Phys. Chem. Chem. Phys.*, 2005, **7**, 3297–3305.
12. F. Weigend, *Phys. Chem. Chem. Phys.*, 2006, **8**, 1057–1065.
13. A. Hellweg, C. Hättig, S. Höfener and W. Klopper, *Theor. Chem. Acc.*, 2007, **117**, 587–597.
14. F. Neese, *WIREs Comput. Mol. Sci.*, 2012, **2**, 73–78.
15. F. Neese, *WIREs Comput. Mol. Sci.*, 2018, **8**, e1327.
16. S. Grimme, S. Ehrlich and L. Goerigk, *J. Comput. Chem.*, 2011, **32**, 1456–1465.
17. V. Barone and M. Cossi, *J. Phys. Chem. A*, 1998, **102**, 1995–2001.
18. B. de Souza, G. Farias, F. Neese and R. Izsák, *J. Chem. Theory Comput.*, 2019, **15**, 1896–1904.
19. B. de Souza, F. Neese and R. Izsák, *J. Chem. Phys.,* 2018, **148**, 34104.
20. M. D. Hanwell, D. E. Curtis, D. C. Lonie, T. Vandermeersch, E. Zurek and G. R. Hutchison, *J. Cheminform.*, 2012, **4**, 17.
